# LHPP Attenuates Lipid Dysfunction of Uveal Melanoma by Relieving the Histidine Phosphorylation of ACO2

**DOI:** 10.34133/research.0896

**Published:** 2025-09-22

**Authors:** Zhi Yang, Liang Ma, Yidian Fu, Xiaoyu He, Yingxiu Luo, Shengfang Ge, Renbing Jia, Jian Huang, Xianqun Fan

**Affiliations:** ^1^ State Key Laboratory of Eye Health, Department of Ophthalmology, Ninth People’s Hospital, Shanghai Jiao Tong University School of Medicine, Shanghai 200011, China.; ^2^ Shanghai Key Laboratory of Orbital Diseases and Ocular Oncology, Shanghai 200011, China.; ^3^ Department of Biochemistry and Molecular Cell Biology, Shanghai Key Laboratory of Tumor Microenvironment and Inflammation, Shanghai Jiao Tong University School of Medicine, Shanghai 200025, China.

## Abstract

Histidine phosphorylation, the neglected but vital phosphoproteome, is a reversible posttranslational modification catalyzed by histidine kinases and erased by phosphohistidine (pHis) phosphatases (e.g., LHPP). Traditional types of phosphorylation have been implicated with the deadliest adult ocular tumor uveal melanoma (UM), which is lipid metabolism dysfunction related. However, the role of histidine phosphorylation in UM remains unknown. Here, up-regulated histidine phosphorylation is associated with poor UM prognosis. Reversal of histidine phosphorylation by LHPP exerts therapeutic effects. Mechanistically, we identified LHPP as metabolically related protein with mitochondrial targeting sequence. LHPP interacts and reduces excessive histidine phosphorylation of mitochondrial aconitase ACO2 at H73 site, thus restoring ACO2 enzymatic activity and mitochondrial citrate transit in TCA cycle. Reduction of citrate accumulation attenuates overloaded lipid synthesis. Besides, genetic ablation of LHPP in mouse eye exhibits abnormal lipid metabolism. These findings illustrate the antagonistic roles of oncogenic histidine phosphorylation and therapeutic mitochondrial LHPP and provide metabolic insight of pHis modification in ocular diseases.

## Introduction

Phosphohistidine (pHis, pH) is one of the 9 known phosphorylations of the 20 natural amino acids in proteins. The 3 canonical phosphorylation sites, i.e., serine (pSer, pS), threonine (pThr, pT), and tyrosine (pTyr, pY), have been extensively studied. In addition, the 6 overlooked hidden phosphoproteomes, i.e., histidine (pHis, pH), lysine (pLys, pK), arginine (pArg, pR), cysteine (pCyS, pC), aspartate (pAsp, pD), and glutamate (pGlu, pE), are poorly understood [1]. pHis was first discovered in 1962 by Paul Boyer, who reported its presence in the mitochondrial enzyme succinyl coenzyme A (CoA) synthase (SCS or SUCLG1), which generates guanosine triphosphate (GTP) in the tricarboxylic acid (TCA) cycle [2]. pHis is a vital modification in prokaryotes and is estimated to account for as much as 6% of all phosphorylated amino acids in eukaryotes [3]. Due to its chemical instability (heat and acid labile), pHis has been studied relatively little in the last half century until recently, when pHis-selective antibodies [4–6] became available and standardized pHis signal detection protocols were developed [7]. pHis has 2 isoforms, 1-pHis and 3-pHis, corresponding to phosphorylation at the 1-nitrogen (N1) or 3-nitrogen (N3), respectively, on the histidine imidazole ring [8,9]. In mammalian cells, histidine can be phosphorylated by 2 histidine kinases, NME1 and NME2 [1,8,10]. In contrast, 3 known pHis phosphatases, namely, phospholysine phosphohistidine inorganic pyrophosphate phosphatase (LHPP), PHPT1, and PGAM5, can dephosphorylate pHis [11]. Histidine phosphorylation plays multiple roles in cellular processes, including cell cycle progression [4], cell migration and protein translation [12], and macromolecular metabolism [13]. Recently, increased pHis protein levels were observed in both hepatocellular carcinoma [8] and pediatric neuroblastoma [12], which uncovered the new chapter of pHis function in tumorigenesis.

LHPP, also called HDHD2B, is a histidine phosphatase that broadly acts on N3-phosphorylated proteins, but no specific pHis substrates of LHPP have been identified [8]. As one of the only 3 known pHis phosphatases, LHPP has long been recognized as a risk gene only for major depressive disorder [13,14].In 2018, Hall and Hunter first reported that LHPP, a tumor suppressor in liver cancer, is triggered by phosphatidylinositol 3-kinase (PI3K)–AKT phosphorylation (pSer) [8]. Thereafter, LHPP has become a popular topic in tumor research [15]. LHPP has been suggested to play a tumor suppressor role in various cancers, while the molecular mechanism identified in most subsequent studies was attributed to the AKT-pSer pathway, which regulates tumor cell proliferation, metastasis, and apoptosis. Additionally, the histidine phosphatase activity of LHPP and protein pHis levels have rarely been discussed.

Uveal melanoma (UM) is the most common primary intraocular malignancy in adults [16,17]. As the second most common type of melanoma, UM accounts for approximately 5% of all melanoma diagnoses, with a high rate of recurrence and poor prognosis [16]. Almost 50% of patients develop metastatic disease in the liver, which can be fatal within 1 year. UM development is related to tumor cell lipid metabolism. In the early diagnosis of small UMs, autofluorescence photography is used by detecting lipofuscin, which is an insoluble brown pigment that over 50% contents are lipids [17]. Unlike cutaneous melanoma, which usually harbors NRAS or BRAF mutations [17], UM is usually initiated by an oncogenic mutation in GNAQ (43%) or GNA11 (49%) [18], which results in constitutive activation of multiple downstream signaling cascades, including the PI3K–AKT, mitogen-activated protein kinase (MAPK), and yes-associated protein 1 (YAP1) pathways [18], thus stimulating abnormal cell proliferation. Key phosphorylated molecules include AKT (pSer^473^) [19], MAPK kinase 1/2 (MEK1/2) (pSer^217/221^), extracellular signal–regulated kinase 1/2 (ERK1/2) (pThr^202^/pTyr^204^) [20,21], YAP1 (pTyr^357^), and focal adhesion kinase (FAK) (pTyr^397^) [22–24]. To date, all the studies on this topic have focused on 3 canonical phosphorylation types (serine, threonine, and tyrosine), but none of the pHis-modified proteins have been reported.

The lack of knowledge about histidine phosphorylation and the pHis phosphatase LHPP in UM, together with the intimate association of constitutive protein phosphorylation with UM, led us to demonstrate the enigmatic function and potential molecular mechanism of histidine phosphorylation in UM tumorigenesis.

In this study, we demonstrated that the global histidine phosphorylation level in UM is aberrantly increased in human patient samples and indicates an unfavorable prognosis. In contrast, low LHPP expression is observed in UM and implies a poor prognosis. Removal of histidine phosphorylation by LHPP suppressed UM growth both in vitro and in vivo. Mechanistically, mitochondrial LHPP interacts with citrate production-related mitochondrial aconitase 2 (ACO2) [25–27] and decreases the pHis of ACO2 H73. Eliminating citrate accumulation by LHPP attenuates disordered lipid synthesis and ameliorates mitochondrial dysfunction. Taken together, we expounded the oncogenic role of histidine phosphorylation in UM and clarified the tumor suppressor and metabolic guarder roles of mitochondrial LHPP in UM.

## Results

### High histidine phosphorylation is a risk factor in ocular melanoma

**Fig. 1.**
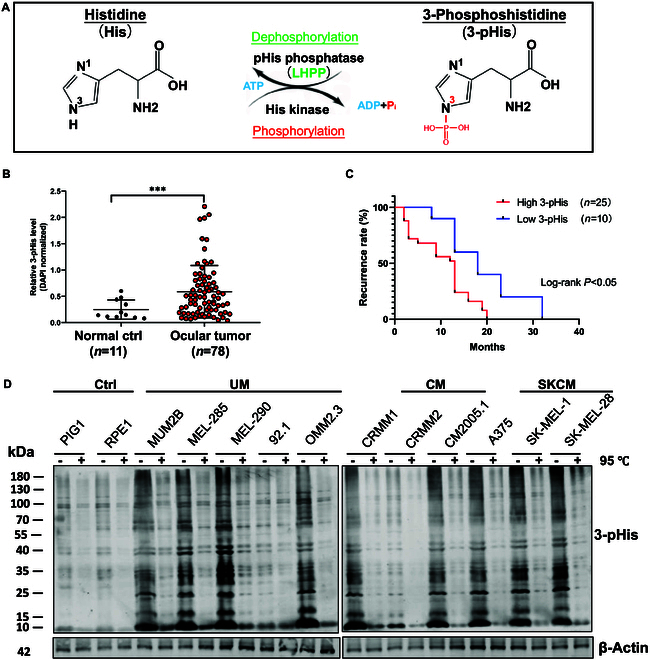
Histidine phosphorylation is up-regulated in ocular melanoma. (A) Schematic diagram: Process of histidine phosphorylation by histidine kinases and dephosphorylation by a 3-pHis phosphatase (LHPP). (B) Quantification of relative 3-pHis levels in human ocular normal ctrl tissues (*n* = 11) and ocular melanoma tissue (*n* = 78) by IF staining. Unpaired *t* test with Welch’s correction. ***P < 0.001. (C) Kaplan–Meier curves of tumor recurrence showing the difference between ocular melanoma patients with low (*n* = 10) and high (*n* = 25) 3-pHis. Log-rank test, *P* < 0.05. (D) Western blotting: 3-pHis level in the control (ctrl) group of normal melanocytes, UM cells, CM cells, and SKCM cells. β-Actin was used as an internal reference protein.

Histidine (His) phosphorylation is a reversible protein modification with 2 isoforms, 3-pHis and 1-pHis. Among known pHis enzymes, the proportion of the 3-pHis isoform appears to be higher than 1-pHis isoform [1]. Histidine phosphorylation is catalyzed by histidine kinases and reversed by pHis phosphatases, especially LHPP, which broadly acts on N3-pHis in the histidine imidazole ring [8,28]. For the phosphorylation process, histidine kinases transfer γ-phosphate from adenosine triphosphate (ATP) onto N3 in the imidazole ring of histidine residues in proteins, while for the dephosphorylation process, pHis phosphatases such as LHPP catalyze the hydrolysis of the phosphate from N3 in the histidine imidazole ring to release a free phosphate ion (Pi) (Fig. 1A). To assess the global protein 3-pHis level in UM, we analyzed a tissue chip embedded with 11 human normal ocular tissues and 78 human ocular melanoma tissues (Table S1). Overall, the relative 3-pHis level was markedly elevated in the UM group (Fig. 1B). Furthermore, a higher 3-pHis indicated earlier recurrence (Fig. 1C). Consistent with these findings, increased 3-pHis levels were found in most melanoma cell lines, including skin cutaneous melanoma (SKCM), conjunctival melanoma (CM), and UM cell lines, especially the UM cell line Mum2b (Fig. 1D). In brief, these data indicate that an increased level of histidine phosphorylation is a risk factor for UM.

Considering the critical role of phosphatase LHPP in histidine dephosphorylation, we subsequently evaluated the expression of LHPP. We performed a single-cell RNA sequencing (scRNA-seq) reanalysis of 6 UM primary tumors [29]. UM is a highly metastatic cancer, and preferentially expressed antigen in melanoma (PRAME) was identified as an oncogene and biomarker for metastasis [29]. Here, a subcluster of PRAME-positive UM cells was annotated (Fig. S1A). Among the 39,312 UM tumor single cells, only 524 LHPP-positive UM tumor cells were annotated (Fig. S1B). Moreover, a low level of histidine phosphatase LHPP was correlated with high PRAME expression in PRAME-positive UM tumor cells, but opposite phenomenon as high level of histidine kinase (NME1 and NME2) (Fig. S1C), which indicates that low LHPP expression forebodes a risk of easier metastasis. Single-cell resolution data imply that loss of LHPP is a poor prognostic characteristic of UM.

Contrary to the increase in 3-pHis modification, the LHPP protein level was dramatically decreased in the tissues of the UM patient group (Fig. S1D). Lower protein LHPP levels in patient tissues (Fig. S1E) indicated a poor prognosis. Both the protein (Fig. S1F) and mRNA (Fig. S1G) levels of LHPP were clearly reduced in most melanoma cell lines. Taken together, these findings demonstrate that LHPP deficiency is a common marker in multiple cancers and is strongly correlated with unfavorable prognosis in UM patients.

### LHPP acts as a tumor suppressor in UM

**Fig. 2.**
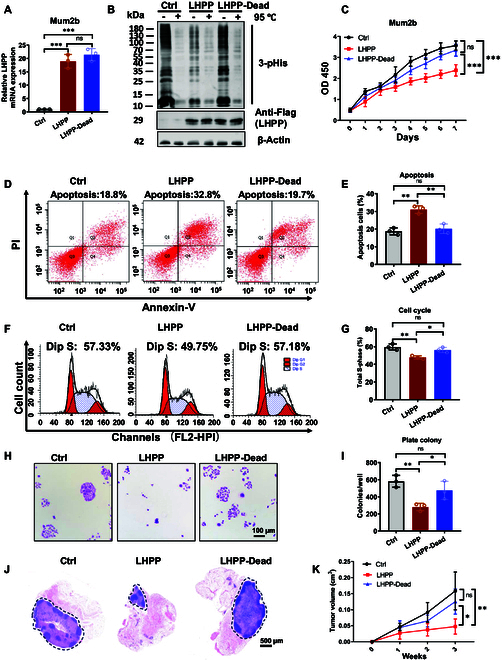
Overexpression of LHPP inhibits UM growth both in vitro and in vivo. (A) Relative LHPP mRNA level in Mum2b cells with stable LHPP and LHPP-Dead overexpression, measured by RT-qPCR. *n* = 3. One-way ANOVA with Tukey’s post hoc test. ***P < 0.001. (B) LHPP protein and 3-pHis levels in Mum2b cells (C) UM cell proliferation assay, as evaluated by a cell counting kit-8 (CCK-8) assay. *n* = 3. One-way ANOVA with Tukey’s post hoc test. **P < 0.01. (D) Apoptosis detection by fluorescence-activated cell sorting (FACS) and (E) statistical analysis in Mum2b cells. *n* = 3. One-way ANOVA with Tukey’s post hoc test. **P < 0.01. (F) Cell cycle analysis by FACS and (G) statistical results. *n* = 3. One-way ANOVA with Tukey’s post hoc test. ***P < 0.001. (H) A colony formation assay and (I) quantification of visible colonies were performed to assess the growth of ocular melanoma cells (based on Mum2b cells). Scale bar, 100 μm. *n* = 3. One-way ANOVA with Tukey’s post hoc test. **P < 0.01. (J) Tumors from subcutaneous xenograft mouse models. (Left) Digital image. (Right) Hematoxylin and eosin (H&E) staining of tumors. Scale bar, 500 μm. (K) Analyses of tumor growth and volume. BALB/c nude mice, *n* = 6. One-way ANOVA with Tukey’s post hoc test. ***P < 0.001.

To evaluate whether the high 3-pHis level and low LHPP level are correlated with tumorigenesis in UM, we next investigated whether LHPP catalytic activity is essential for the above phenomenon. LHPP is a haloacid dehalogenase (HAD) family phosphatase, which is as an Asp (D)-dependent hydrolase. Double mutations of D17 and D214 have been used to block LHPP phosphatase catalytic activity [14,30]. We subsequently established a Flag-tagged LHPP and phosphatase-inactive LHPP variant (double mutation of D17N and D214N, called LHPP-Dead)-overexpressing UM Mum2b cells by lentiviral transduction. Exogenous overexpression of LHPP and LHPP-Dead mRNA was further confirmed (Fig. 2A). Moreover, exogenous LHPP decreased the 3-pHis signal, while LHPP-Dead showed no obvious difference (Fig. 2B). Consistently, similar phenomenon was observed in the immunofluorescence (IF) result (Fig. S2A and B). Furthermore, double mutation of D17N and D214N in LHPP indeed reduced its phosphatase activity in a pNPP phosphatase assay (Fig. S2C). According to the LHPP protein quaternary structure [Protein Data Bank (PDB): 2X4D], Asp^17^ and Asp^214^ lie 2.97 Å apart, forming a rich hydrogen bond network (Fig. S2D), which suggests that D17N/D214N double mutation may disrupt this network and thus impact LHPP phosphatase activity. Here, LHPP effectively inhibited UM cell proliferation, whereas LHPP-Dead did not have a marked inhibitory effect (Fig. 2C), which indicates that the suppressor effect of LHPP is phosphatase catalytic activity dependent. Besides, LHPP wild-type (WT), but not LHPP-Dead, increased apoptosis (Fig. 2D and E) and modestly decreased the S-phase population of UM cells (Fig. 2F and G). Moreover, LHPP WT prominently suppressed cell colony formation (Fig. 2H and I). These results demonstrated the suppression of cell proliferation by LHPP in vitro. In subcutaneous xenograft mouse model, LHPP WT effectively suppressed tumor growth (Fig. 2J) and resulted in a decreased tumor volume (Fig. 2K). Thus, the expression of LHPP WT prevents UM cell proliferation and tumor growth both in vitro and in vivo, suggesting that the protein histidine phosphatase LHPP functions as a tumor suppressor in the rare ocular disease UM.

### The aconitase ACO2 is a potential substrate of mitochondrial LHPP

To further understand the underlying molecular mechanism that LHPP functions in UM, we next analyzed the potential functional partners of human LHPP using the protein network prediction database STRING and the results suggested that the inorganic pyrophosphatase (PPA) family and several ATPases closely interact with LHPP (Fig. S3A). Specifically, Oxidative phosphorylation and Metabolic pathway were the 2 most associated pathways (Fig. S3B). To investigate the potential downstream targets of LHPP at the transcriptional level, we performed RNA-seq analysis of Mum2b cells stably overexpressing LHPP. The top 5 up-regulated genes were AMOT, LHPP, ZNF608, TENM2, and CDK15 (Fig. S3C). Interestingly, the differentially expressed genes were enriched mainly in metabolism-related Kyoto Encyclopedia of Genes and Genomes (KEGG) pathways, including Metabolic pathways, Histidine metabolism, and Pathways in cancer (Fig. S3D). Because protein–protein interactions (PPIs) are essential for phosphatase function, we next investigated the potential interacting proteins of LHPP. Thus, we performed immunoprecipitation (IP) to obtain samples for mass spectrometry analysis by using Flag-LHPP as bait protein in UM Mum2b cells. The top enriched proteins, LHPP (rank 1) and ACO2 (rank 2), were highlighted (Fig. S3E). Consistently, KEGG pathway analysis showed several metabolism pathway enrichments, including the metabolic and histidine metabolism pathways (Fig. S3F), indicating a possible connection between LHPP-partner proteins and metabolic processes. Accordingly, these results suggest that LHPP is a key protein that modulates metabolic processes in UM cells.

**Fig. 3.**
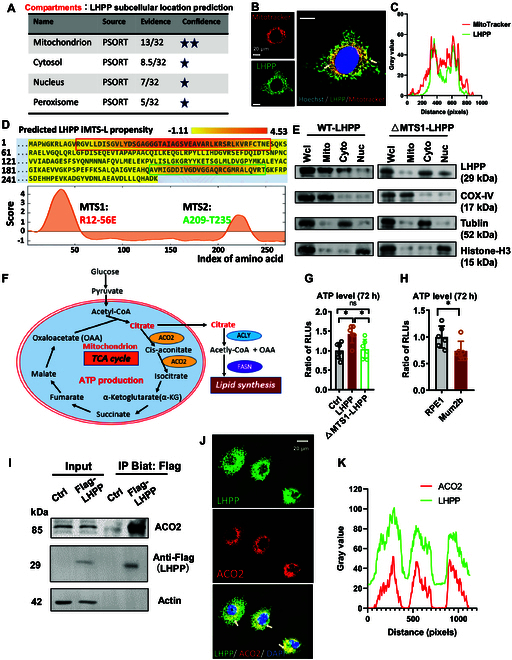
LHPP interacts with ACO2 in mitochondria. (A) The subcellular location of LHPP was predicted by COMPARTMENTS. (B) Subcellular IF staining of LHPP in UM Mum2b cells stably overexpressing LHPP. Counterstaining for endogenous LHPP (green), the mitochondrial marker MitoTracker (red), and nuclear marker Hoechst (blue). Scale bar, 20 μm. (C) Signal colocalization analysis: A rectangle scan of the relative fluorescence intensity of the signal is plotted to show the peak overlap of LHPP (green) and MitoTracker (red). (D) Prediction of putative MTS of human LHPP protein by iMLP. (E) Identification of the subcellular location of LHPP protein in UM Mum2b cell line stably overexpressing WT-LHPP or ΔMTS1-LHPP. (F) Graphical illustration: Mitochondrial ACO2 controls citrate production in the TCA cycle for cytoplasmic lipid synthesis. (G) ATP levels in UM Mum2b control, LHPP, and ΔMTS1-LHPP-overexpressing cells. *n* = 6. Two-tailed unpaired Student’s *t* test. ***P < 0.001. (H) ATP level measurement in UM Mum2b cells and normal RPE1 cells. *n* = 6. Two-tailed unpaired Student’s *t* test. *P < 0.05. (I) Co-IP analysis of the interaction between LHPP and ACO2 in Mum2b control and Mum2b LHPP-Flag-overexpressing cells. (J) IF staining showing the colocalization of LHPP and mitochondrial protein ACO2 in UM Mum2b cells stably overexpressing LHPP. Scale bar, 20 μm. (K) Signal colocalization analysis: A rectangle scan of the relative fluorescence intensity of the signal is plotted to show the peak overlap of LHPP (green) and ACO2 (red).

As mitochondrion is a major organelle responsible for metabolism, we next analyzed the localization of the human LHPP protein with the subcellular localization prediction tool COMPARTMENTS and found that LHPP could localize in the mitochondrion (Fig. 3A). In UM Mum2b cells, immunocytochemical (ICC) staining revealed that LHPP was mildly localized to the cytosol and nucleus but was enriched primarily in mitochondria. LHPP exhibited clear overlap with the mitochondrial marker MitoTracker (Fig. 3B and C). Cyclic guanosine monophosphate (GMP)–adenosine monophosphate (AMP) synthase (cGAS), a known cytosolic and nuclear protein, was recently reported in the mitochondrial fraction of Hep3B cells due to the mitochondrial targeting sequence (MTS) within cGAS [31]. We next analyzed the potential MTS in the human LHPP protein by MTS analysis tool iMPL. Here, 2 putative MTSs of LHPP were identified, one from residues 12 to 56 (MTS1) and one from residues 209 to 235 (MTS2) (Fig. 3D). MTS1 and MTS2 were fused with green fluorescent protein (GFP) individually, and results showed that only fusion of LHPP 12–56 (MTS1) targeted GFP to the mitochondria in Mum2b cells (Fig. S4A). Human LHPP protein structure (PDB: 2X4D) analysis indicated that LHPP 12–56 residues were close to the N terminus and mainly localized on the surface of the protein (Fig. S4B). Then, we isolated mitochondrial, cytosolic, and nuclear fractions from equal numbers of Mum2b cells and observed that both exogenous LHPP protein (Fig. 3E, left) and endogenous LHPP protein (Fig. S4C) were enriched in the mitochondrial fraction. Mitochondrial distribution of the LHPP mutant that lacked the predicted MTS1 (12–56 in LHPP, named ΔMTS1-LHPP) was decreased in UM Mum2b cells (Fig. 3E, right), indicating that the predicted protein MTS1 was required for LHPP localization to mitochondria. Taken together, the above results suggest that LHPP is a protein with mitochondrial location in UM cells.

Mitochondrial aconitate hydratase 2, also called aconitase 2 (i.e., ACO2), is one of the enriched proteins identified by protein mass spectrometry after IP with Flag-LHPP. ACO2 protein was enriched with a 32-fold increase in the Flag-LHPP group, and LHPP protein was relatively abundant, with a 462-fold increase (Fig. S3E). ACO2 is a reversible citrate hydrolyase in mitochondria that catalyzes the interconversion of mitochondrial citrate to isocitrate in the second step of the TCA cycle [26,32,33]. Mitochondrial citrate is the precursor metabolite required for lipogenesis and is exported to the cytosol for conversion into acetyl-CoA by ATP-citrate lyase (ACLY) for de novo biosynthesis of fatty acids [34]. In tumors, the demand for de novo lipogenesis is increased, which is associated with increased levels of rate-limiting enzymes such as fatty acid synthase (FASN) [35]. In brief, ACO2 acts as a key hub in citrate-linked lipid metabolism (Fig. 3F).

Metabolic adaptation is one of the essential cancer hallmarks for maintaining replication and survival stress [36]. Dysregulation of lipid metabolism is a hallmark of cancer progression [27]. According to the Warburg effect, due to mitochondrial dysfunction, cancer cells prefer aerobic glycolysis for less ATP generation rather than mitochondrial respiration [37]. Based on nutrient conditions, tumor cells switch metabolic pathways for mitochondrial ATP synthesis [27]. To investigate whether the antitumor effect of LHPP is mediated via the alteration of ATP generation in tumor cells, we measured the ATP level in UM Mum2b cells after LHPP overexpression. LHPP WT, but not ΔMTS1-LHPP, dramatically increased the ATP level in UM cells (Fig. 3G), indicating partial restoration of mitochondrial function. Alternatively, the ATP concentration in UM Mum2b cells was lower than that in normal RPE1 cells (Fig. 3H).

These results led us to ask whether LHPP changes ATP generation by directly targeting the key regulator ACO2 in the TCA cycle. To determine whether there is indeed a direct interaction between the mitochondrial LHPP protein and ACO2 in UM cells, we performed a co-IP assay to confirm the direct PPI. Here, ACO2 was abundantly detected after IP with Flag-LHPP (Fig. 3I). In addition, we further observed a distinct overlap of human LHPP and ACO2 protein signals by immunocytochemistry in UM Mum2b cells (Fig. 3J and K).

Taken together, these results indicate that the LHPP protein has a mitochondrial function in UM cells and directly physically interacts with another mitochondrial enzyme, ACO2.

### The enzymatic activity of ACO2 is dependent on H73 histidine phosphorylation

We used the posttranslational modification (PTM) tool PhosphoSitePlus to analyze PTM sites in the human ACO2 protein, including abundant phosphorylation, acetylation, and ubiquitylation sites (Fig. S5A). However, the phosphorylation types include the 3 canonical phosphorylations but without pHis information of ACO2. HisPhosSite [3] is a comprehensive database of pHis-modified proteins launched in 2021. We analyzed the experimentally verified classical histidine phosphorylation-related proteins [9], including NME1, NME2, PGAM1, GNB1, and SUCLG1 (SCS), in *Homo sapiens* and the predicted pHis sites in *Mus musculus* or *Rattus norvegicus*. The verified pHis sites in *H. sapiens* were consistent with previously reported results [9], while no pHis sites in the human ACO2 protein were found (Fig. S5B). Recently, a strong cation exchange (SCX) chromatography enrichment method was developed to reveal the histidine phosphoproteome [38]. Here, 563 different pHis peptides were identified among 385 proteins, including the abovementioned 5 classic pHis proteins. Interestingly, human ACO2 protein was identified, with a pHis modification at H73, which was identified with a specific peptide (IVYGHLDDPASQEIER) (Fig. S5C).

**Fig. 4.**
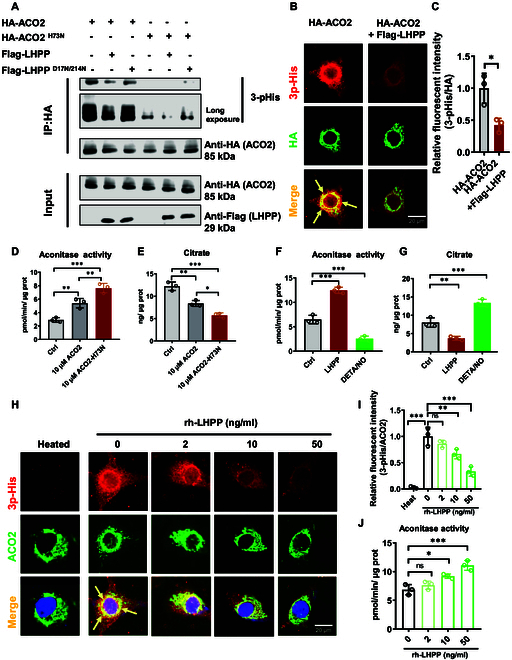
LHPP regulates histidine phosphorylation and enzymatic activity of ACO2. (A) Co-IP assay to evaluate the 3-pHis level of ACO2 in UM Mum2b cells. (B) Immunocytochemical counterstaining and (C) quantification of ACO2 and 3-pHis in UM Mum2b cells with or without coexpression of HA-ACO2 and Flag-LHPP. Scale bar, 20 μm. Two-tailed unpaired Student’s *t* test. *P < 0.05. (D) Activity of aconitase and (E) citrate concentration in UM Mum2b cells treated with 10 μM human recombinant ACO2 or ACO2 H73N proteins. *n* = 3. One-way ANOVA with Tukey’s post hoc test. *P < 0.05. **P < 0.01. (F) Activity of aconitase and (G) citrate concentration in UM Mum2b cells stably overexpressing LHPP or treated with DETA/NO (500 μM). *n* = 3. One-way ANOVA with Tukey’s post hoc test. *P < 0.05, **P < 0.01, ***P < 0.001. (H) IF costaining and (I) quantification of 3-pHis and ACO2 in Mum2b cells after different concentration of recombinant human LHPP protein treatment. Data were analyzed by one-way ANOVA with Tukey’s post hoc test. **P < 0.01, ***P < 0.001. (J) Activity of aconitase in UM Mum2b cells treated with different rh-LHPP protein. Data were analyzed by one-way ANOVA with Tukey’s post hoc test. *P < 0.05, ***P < 0.001. *n* = 3.

In 2016, a report revealed another histidine phosphatase, PGAM5, that specifically dephosphorylated NME2 (also called NDPK-B) on H118 but failed to dephosphorylate NME2 after NME2 H118 was mutated to asparagine (H118N) [39]. We next constructed human ACO2 WT and an ACO2 H73N mutant designed to block His H73 phosphorylation. Thereafter, we detected the LHPP-mediated dephosphorylation of ACO2 by coexpression of ACO2 WT, ACO2 H73N, LHPP WT, and LHPP-Dead in UM Mum2b cells. Phosphorylation of ACO2 on H73 cells was then assessed. Indeed, LHPP WT, but not LHPP-Dead, reduced the pHis level of ACO2. However, compared with ACO2 WT, the pHis level of ACO2 H73N was clearly lower (Fig. 4A). In addition, ICC staining of UM Mum2b cells revealed overlap of 3-pHis and ACO2 signals, and LHPP suppressed the 3-pHis level of ACO2 (Fig. 4B and C). Taken together, these results suggest that LHPP decreases the histidine phosphorylation of ACO2 at H73.

In addition, blockade of ACO2 histidine phosphorylation by the H73N mutation (treated with purified recombinant ACO2 H73N protein) increased the aconitase activity (Fig. 4D) but decreased mitochondrial citrate accumulation (Fig. 4E) in UM Mum2b cells. DETA/NO is a reported ACO2 inhibitor that can arrest cells and substantially reduce ACO2 enzymatic activity [26]. Interestingly, treatment with the positive control DETA/NO decreased ACO2 enzymatic activity, while LHPP overexpression efficiently increased the aconitase activity (Fig. 4F). Conversely, the level of the key metabolite citrate was reduced after LHPP overexpression and increased after DETA/NO treatment (Fig. 4G).

To further confirm that LHPP can dephosphorylate ACO2 via biochemical assays, we utilized recombinant human LHPP (rh-LHPP) protein (0, 2, 10, or 50 ng/ml) to test the in vitro dephosphorylation efficiency. Here, a visibly lower 3-pHis level (IF) was observed in the 50 ng/ml rh-LHPP-treated group. The 3-pHis (red)–ACO2 (green) merged signal (orange, considered histidine-phosphorylated ACO2) was consistently decreased (Fig. 4H and I). In contrast, downstream aconitase activity was increased after 50 ng/ml rh-LHPP treatment (Fig. 4J). In short, H73 is one of the key 3-pHis-modified residues of ACO2 in UM. LHPP decreases the histidine phosphorylation level of ACO2 at H73, which enhances the enzymatic activity of ACO2 and subsequently enhances mitochondrial citrate metabolism.

Taken together, we demonstrated that H73 is one of the key 3-pHis-modified residues of ACO2 in UM. LHPP decreases the histidine phosphorylation level of ACO2 at H73, which enhances the aconitase activity and subsequently enhances mitochondrial citrate metabolism.

### ACO2 histidine phosphorylation is critical for LHPP-mediated antitumor effects

**Fig. 5.**
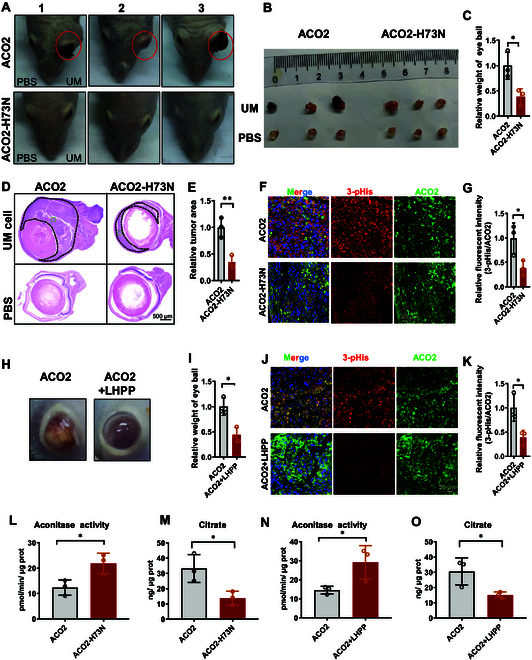
LHPP prohibits UM growth by reducing histidine phosphorylation of ACO2. (A) Representative images of the UM intraocular orthotopic xenograft mouse model established with UM Mum2b cells with stable ACO2 or ACO2 H73N expression. Phosphate-buffered saline (PBS) was administered to the other eye of each mouse as a negative control. *n* = 3. (B) Representative images of in vivo ocular tumor formation in mice. (C) Statistical analysis of the tumor weight. Two-tailed unpaired Student’s *t* test. *P < 0.05. *n* = 3. (D) H&E staining of UM intraocular orthotopic xenografts. The ocular tumor is outlined with a black dotted line. Scale bar, 500 μm. (E) Statistical analysis of the tumor area. Two-tailed unpaired Student’s *t* test. **P < 0.01. *n* = 3. (F) IF counterstaining of ACO2 and 3-pHis in the tumor outlined with a green dotted line in (D). Scale bar, 20 μm. (G) Statistical analysis of the relative level of 3-pHis (normalized to total ACO2). Two-tailed unpaired Student’s *t* test. *P < 0.05. *n* = 3. (H) Representative images of the UM intraocular orthotopic xenograft mouse model established with UM Mum2b cells expressing ACO2 or coexpressing ACO2 and LHPP. (I) Statistical analysis of tumor weight. Two-tailed unpaired Student’s *t* test. *P < 0.05. *n* = 3. (J) IF counterstaining of ACO2 and 3-pHis in tumors in the model established with UM Mum2b cells expressing ACO2, or coexpressing ACO2 and LHPP. Scale bar, 200 μm. (K) Quantification of the relative level of 3-pHis ACO2 (normalized to total ACO2). Two-tailed unpaired Student’s *t* test. *P < 0.05. *n* = 3. (L) Enzymatic activity of aconitase and (M) citrate concentration in UM intraocular orthotopic xenograft mouse eye tumor tissues with stable ACO2 or ACO2 H73N overexpression in UM Mum2b cells. Two-tailed unpaired Student’s *t* test. *P < 0.05. *n* = 3. (N) Enzymatic activity of aconitase and (O) citrate concentration in UM intraocular orthotopic xenograft mouse eye tumor tissues with UM Mum2b cells with stable ACO2 expression, or ACO2 and LHPP coexpression. Two-tailed unpaired Student’s *t* test. *P < 0.05. *n* = 3.

In 2022, ACO2 was reviewed as a potential prognostic and immunotherapeutic biomarker in a pan-cancer analysis study, where ACO2 was highly expressed in most cancers but was either positively or negatively associated with prognosis in different tumors [40]. These findings led us to examine the potential role of ACO2 in UM tumorigenesis. We further investigated the potential oncogenic function of the ACO2 pHis73 PTM. Interestingly, expression of ACO2 H73N showed a distinct inhibition of tumor growth in the intraocular xenograft mouse model (Fig. 5A). Blockade of histidine phosphorylation of ACO2 by the H73N mutation resulted in smaller size and lower weight of ocular tumors (Fig. 5B and C). Besides, representative pathological results showed lower ocular tumor occupancy in ACO2 H73N group mice (Fig. 5D and E). Additionally, IF staining of the enclosed mouse tumor tissues revealed a decrease in the 3-pHis level and overlap with ACO2, suggesting the decrease of ACO2 histidine phosphorylation (Fig. 5F and G). Similarly, blockade of ACO2 histidine phosphorylation by the pHis phosphatase LHPP exhibited a similar therapeutic effect (Fig. 5H and I), which was attributed to the lower 3-pHis level of ACO2 (Fig. 5J and K). In summary, silencing the histidine phosphorylation of ACO2 via the H73N mutation or the pHis phosphatase LHPP inhibited UM tumor growth in vivo.

Consistent with the in vitro results, blockade of ACO2 histidine phosphorylation by the H73N mutation increased the enzymatic activity (Fig. 5L) but reduced citrate accumulation (Fig. 5M) in the eye tissue of xenograft mice. Moreover, blockade of ACO2 histidine phosphorylation by the pHis phosphatase LHPP increased the aconitase activity (Fig. 5N). In contrast, the citrate concentration decreased after LHPP overexpression (Fig. 5O).

Taken together, these findings support the concept that excessive pHis phosphorylation of ACO2 enhances tumor growth. Blocking the histidine phosphorylation of ACO2 by the H73N mutation and the pHis phosphatase LHPP prevents UM tumor growth.

### Loss of LHPP triggers abnormal lipid metabolism in UM

**Fig. 6.**
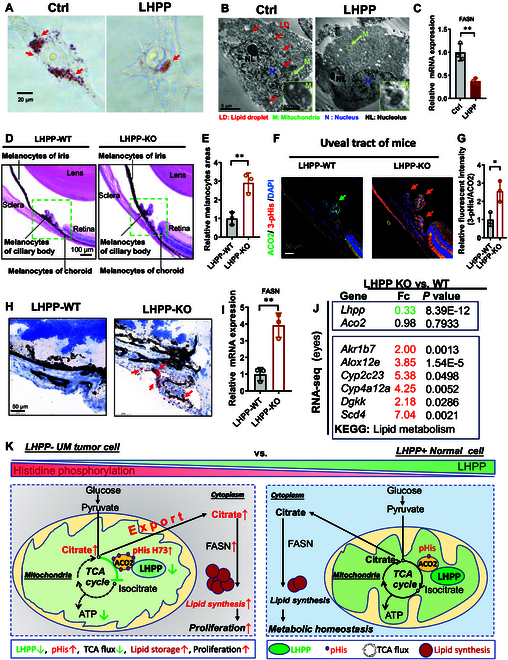
LHPP-pHis ACO2–citrate axis restores cellular lipid metabolism in UM. (A) Oil Red O staining of UM Mum2b cells stably overexpressing LHPP. Lipid accumulation was visualized as red droplets. Scale bar, 20 μm. (B) Representative scanning transmission electron micrograph of UM Mum2b cells stably overexpressing LHPP. Scale bar, 5 μm. Images of mitochondrial morphology were magnified. Scale bar, 500 nm. N, nucleus; NL, nucleolus; LD, lipid droplet; M, mitochondrion. (C) Relative human FASN mRNA expression. Two-tailed unpaired Student’s *t* test. **P < 0.01. (D) H&E image and (E) quantification of the uveal tract in LHPP-WT and LHPP-KO mice. *n* = 3. Scale bar, 100 μm. Two-tailed unpaired Student’s *t* test. **P < 0.01. (F) IF counterstaining of ACO2 and 3-pHis in the uveal tract in LHPP-WT and LHPP-KO C57BL/6JGpt mice. Scale bar, 100 μm. (G) Quantification of the relative level of 3-pHis ACO2 (normalized to total ACO2). *n* = 3. Two-tailed unpaired Student’s *t* test. *P < 0.05. (H) Oil Red O staining in the uveal tract of LHPP-WT and LHPP-KO mice. *n* = 3. Scale bar, 50 μm. (I) Relative ocular FASN mRNA expression in LHPP-WT and LHPP-KO mice was measured by RT-qPCR. Two-tailed unpaired Student’s *t* test. **P < 0.01 (*n* = 3). (J) High-throughput analysis (RNA-seq) of altered lipid metabolism genes in the eyes of LHPP-KO mice. *n* = 3. (K) Graphical abstract delineating the antitumor mechanism of LHPP in UM.

Mitochondrial citrate could export across the mitochondrial membrane to cytosol and further acts as an important precursor for fatty acid synthesis [26]. Metabolic adaptation is a vital cancer hallmark that maintains replication [36], and lipid metabolism imbalance is another hallmark of cancer progression [27]. LHPP administration and ACO2 H73N mutation both resulted in distinct changes in aconitase activity and citrate levels in UM. It remains unclear whether the tumor inhibition effect of LHPP impairs downstream metabolite dysfunction. Interestingly, Oil Red O staining showed that LHPP overexpression decelerated lipid droplet (LD) accumulation in UM cells (Fig. 6A). Similarly, transmission electron microscopy (TEM) revealed an abundance of LDs (red arrow) in control UM cells. Moreover, mitochondrial ultrastructure analysis revealed fragmentation of mitochondrial morphology and swelling with enlarged cristae, indicating altered organelle function. However, the normal morphology of the cristae was readily restored by reexpression of LHPP (Fig. 6B). Besides, cellular triglyceride (TG) content was decreased after LHPP augmentation in UM Mum2b cells (Fig. S6A). Mechanistically, how does LHPP promote lipid metabolism in UM cells? To answer this question, we first found that the concentration of citrate (Fig. S6B) and expression level of FASN (Fig. S6C) in UM Mum2b cells were much higher than those in normal RPE1 cells. In contrast, the relative FASN mRNA expression level was markedly reduced after LHPP was reexpressed in UM Mum2b cells (Fig. 6C). Besides, fatty acid synthase inhibitor GSK2194069 could efficiently suppress human FASN mRNA expression in low LHPP UM Mum2b cells (Fig. S6D), which indicate that Mum2b cells are sensitive to the fatty acid synthesis process.

As indicated in the metabolic map, LHPP may control crosstalk among the 3 individual metabolic pathways, lipid metabolism (FASN), histidine metabolism (l-histidine), and the TCA cycle (ACO2, citrate) (Fig. S6E). Specifically, the LHPP–pHis ACO2–citrate axis may contribute to lipid metabolism. LHPP triggers histidine dephosphorylation of the key TCA cycle enzyme ACO2, which alters citrate accumulation and export, thereafter influencing FASN-based lipid synthesis. Citrate was exported to the cytosol from mitochondria in Mum2b cells for superfluous lipid synthesis and could be attenuated by LHPP.

UMs arise from melanocytes in the pigmented uveal tissues of the eye, and the uveal tract is composed of the ciliary body, iris, and choroid [17]. In clinical practice, the visible lipid-enriched feature was used for early diagnosis of UMs by autofluorescence photography [17]. We further generated an LHPP-knockout (KO) mouse model via a CRISPR gene editing strategy to investigate the potential influence of LHPP and lipid status in the early stage of UM. Interestingly, compared with LHPP-WT mice, LHPP-KO mice tended to exhibit pigmentation with a thicker uveal tract (Fig. 6D and E). This abnormal phenotype could be attributed to the increase of ACO2 3-pHis modification (Fig. 6F and G).

Besides, Oil Red O staining showed that LHPP deficiency promoted LD accumulation in the uveal tract of LHPP-KO mice (Fig. 6H). Moreover, mouse FASN mRNA expression was up-regulated in the eyes of LHPP-KO mice (Fig. 6I), indicating a high demand for lipid synthesis in LHPP-deficient mice. We further collected and analyzed the eye tissues of LHPP-KO mice via RNA-seq. The results revealed ablation of the *Lhpp* gene [fold change (Fc) = 0.33] but no change in Aco2 (Fc = 0.98). Moreover, several other lipid metabolism genes were up-regulated, like Akr1b7, Alox12e, Cyp2c23, Cyp4a12a, Dgkk, and Scd4 (Fig. 6J).

Considering these results collectively, we established a concept that LHPP controls 3-way crosstalk among metabolic pathways, namely, lipid metabolism, histidine metabolism, and the citrate cycle. Genetic ablation of LHPP disrupts the pHis modification level and lipid metabolism status in the uveal tract of mice, which is a potential risk factor for early UM development.

## Discussion

In short, we demonstrated the potential metabolic role of mitochondrial LHPP and pHis in UM oncogenesis (Fig. 6K). To date, the function of histidine phosphorylation in UM tumorigenesis has not been determined. Here, we revealed that global histidine phosphorylation is increased in UM and is associated with unfavorable prognosis. Moreover, pHis phosphatase LHPP level is dramatically decreased, and loss of LHPP is correlated with poor prognosis in UM. LHPP deficiency contributes to the increased pHis level of the aconitase ACO2. A high pHis of ACO2 suppresses ACO2 aconitase activity, which leads to blockade of citrate conversion and reduced ATP production in mitochondria. Thereafter, unconsumable citrate is largely transported to the cytoplasm to provide a source for FASN-mediated excessive lipogenesis, which leads to abnormal lipid accumulation in UM cells. Conversely, reexpression of LHPP readily restores mitochondrial function and attenuates citrate accumulation in UM cells by decreasing global histidine phosphorylation, especially the pHis level, of the aconitase ACO2. These findings demonstrate the therapeutic function of LHPP in UM tumorigenesis.

Here, we highlighted the hidden phosphoproteome from the perspective of histidine phosphorylation (pHis) in UM. In recent decades, constitutive activation of the PI3K–AKT, MAPK, and YAP1 pathways has been considered the major pathogenic mechanism of UM [18]. Molecules modified by the 3 classical types of phosphorylation (pSer, pThr, and pTyr) include AKT (pSer) [19], MEK1/2 (pSer), ERK1/2 (pThr/pTyr) [20,21], YAP1 (pTyr), and FAK (pTyr) [22–24]. Unlike the classical perspective, in this study, modulation of the global pHis level via the pHis phosphatase LHPP is a potential therapeutic strategy for UM. In addition, p-FAK (pTyr^397^) is a key activated molecule in UM. Recently, pHis 58 of FAK was reported to promote proliferation in esophageal squamous cell carcinoma [41] and esophageal cancer [42]. Whether pHis 58 of FAK plays a similar role in UM deserves further investigation.

ACO2, the metabolic master regulator in mitochondria [25–27,43], regulates mitochondrial citrate generation to facilitate de novo lipogenesis, while the enzymatic activity of ACO2 plays dual role in lipogenesis related-cancer progression. In prostate cancer, high acetylation of ACO2 Lys^258^ results in high aconitase activity, which promotes citrate synthesis to support lipogenesis and aggressive behaviors [27]. In colorectal cancer, blockade of ACO2 caused a reduction in TCA cycle intermediates and suppression of mitochondrial oxidative phosphorylation, resulting in an increase in citrate flux for fatty acid and lipid synthesis [33]. However, the role of high histidine phosphorylation of ACO2 in UM is quite different. In UM, a high pHis inhibits ACO2 aconitase activity, which forces the transport of overloaded citrate to the cytosol and facilitates its utilization for excess lipogenesis. These findings indicate that PTMs of ACO2, such as acetylation and histidine phosphorylation, have different or even opposite functions in different cancers. This may depend on the citrate–isocitrate reaction direction, controlled by the reversal of ACO2 enzymatic activity.

Abnormal fatty acid synthesis is a risk feature of cancer progression [44]. Lipid metabolism status may be useful for predicting the prognosis of patients with UM [45]. In the early diagnosis of small UMs, autofluorescence photography is used to detect lipofuscin, an insoluble and lipid-accumulated pigment, where half of the components are incomplete oxidation lipids [17]. BAP1 is a key risk protein in UM [17]. LD storage was visible in BAP1-positive and adipophilin-enriched UM primary tumors [46], supporting lipid dysfunction in BAP1 abnormal UM. BAP1-dependent lipid metabolism is related to the up-regulation of lipogenic pathways [47]. Besides, most BAP1-mutated and BAP1-inactive UMs have a high risk of metastatic relapse [48], indicating that lipid metabolism homeostasis may regulate the progression of UM from primary to metastatic. Currently, the development of lipid metabolism-targeted approaches involving BAP1 in UM is urgently needed [49]. Despite gene therapy and immunotherapy [50,51], targeting metabolism might be another therapeutic approach. In this study, we reclarify the concept that excessive LD storage is an oncogenic factor in UM. This reversible biological process is associated with the pHis modification status of mitochondrial aconitase. Further research is needed to determine whether the abnormal BAP1 proteins exhibit different pHis modification levels and are differentially involved in lipid metabolism regulation in primary or metastatic UM.

Although our research highlights the metabolic role in understanding UM development and progression, several limitations remain. First, the upstream kinase of ACO2 still remains unclear, which deserves further study in the future. Even phosphatase LHPP was proved to dephosphorylate 3-pHis of ACO2. Due to the major mitochondrial sublocalization of ACO2, we hypothesized that local histidine kinases in mitochondria could be candidates, for instance, NEM3 and NME4, which were identified as mitochondrial proteins according to mitochondrial protein database Human MitoCarta3.0 [52]. Second, the identification of pHis is another limitation of this work. We verified H73 as one of the key pHis in ACO2 by mining the human histidine phosphoproteome data [38]. However, it is possible that the LHPP-dependent dephosphorylation effect and enzymatic activity of ACO2 may be restricted not only to ACO2 H73 but also to other pHis sites of ACO2. Comprehensive identification of the pHis sites of human ACO2 via mass spectrometry is needed for future study. Third, primary UM tumors are usually treated by radiotherapy or enucleation. The commonly used UM drugs are universal inhibitors, such as MEK inhibitor cobimetinib and FAK inhibitor defactinib [18]. Considering the unfavorable effect of high pHis modification in UM, the potential perspective of future UM clinical application is exploring the broad-spectrum or specific inhibitors targeting abnormal pHis proteins by high-throughput drug screening.

In brief, this study revealed that high histidine phosphorylation level is a feature of poor prognosis. We identified mitochondrial LHPP as a critical tumor suppressor involved in lipid metabolism. Thus, the pHis phosphatase LHPP might be a potential therapeutic target in UM.

## Materials and Methods

### Animal studies

The animal experiments were approved by the Ninth People’s Hospital, Shanghai JiaoTong University School of Medicine Animal Care and Use Committee (SH9H-2022-A372-SB) and conducted in accordance with the animal policies of Shanghai Jiao Tong University and the guidelines established by the National Health and Family Planning Commission of China. LHPP-KO mice on the C57BL/6JGpt genetic background were generated by the CRISPR/Cas9 gene editing strategy by GemPharmatech Co. Ltd. (Nanjing, China). Based on the structure of the mouse *Lhpp* gene, the region spanning exons 2 to 5 of the Lhpp-201 (ENSMUST00000033241.5) transcript, which contains a 499-base pair coding sequence, was selected as the targeted KO region. All animals were housed in a pathogen-free barrier environment (approximately 40% humidity at 20 °C and a 12-h dark/light cycle). The mice were fed a normal chow diet. The BALB/c nude mice (male, 6 weeks old) used for the subcutaneous and intraocular xenograft assay were purchased from the Shanghai Laboratory Animals Center (Shanghai, China). The animal number is no less than 3 for each group.

### Patient samples

A total of 78 human ocular melanoma tissues and 11 human normal control tissues (choroidal melanin tissues) were collected for analysis from patients of Ninth People’s Hospital, Shanghai JiaoTong University School of Medicine. All experiments were approved by Ninth People’s Hospital, Shanghai JiaoTong University School of Medicine Ethics Committee (SH9H-2022-TK191-1). The histological features of all specimens were evaluated by pathologists according to the standard criteria. The clinicopathological characteristics of the patients with ocular melanoma in the associated samples are listed in Table S1. Individual tissue sections from a tissue array were subjected to 3-pHis and LHPP immunohistochemistry (IHC) detection. Normalized fluorescence signal value under 0.3 was defined as low expression group for Kaplan–Meier curves analysis. Typically, patients who were found with local tumor in the eyes during postoperative reexamination were defined as recurrence, and time was recorded.

### Cell culture

The widely used human normal skin immortalized melanocyte cell line PIG1 cells and immortalized retinal pigment epithelial cells RPE-1 were gifts from the Department of Ophthalmology, Peking University Third Hospital. The human UM cell lines MUM2B, MEL-285, MEL-290, 92.1, and OMM2.3 were kindly provided by J. F. Marshall (Tumor Biology Laboratory, Cancer Research UK Clinical Center, John Vane Science Centre, London, UK). The human CM cell lines CRMM1, CRMM2, and CM2005.1 were kindly supplied by M. J. Jager (Leiden University Medical Center, Leiden, Netherlands). The human SKCM cell lines A375 [American Type Culture Collection (ATCC), CRL-1619], SK-MEL-1 (ATCC, HTB-67), and SK-MEL-28 (ATCC, HTB-72) and the human embryonic kidney cell line HEK293T (ATCC, CRL-3216) were purchased. All cell lines used in this project were authenticated by short tandem repeat (STR) profiling. Unless otherwise stated, the cells were cultured in Dulbecco’s modified Eagle’s medium (Gibco). PIG1 and CM2005.1 cells were cultured in RPMI 1640 medium (Gibco). CRMM1 and CRMM2 cells were cultured in Ham’s F-12K medium (Gibco). All media were supplemented with 10% fetal bovine serum (FBS; Gibco) and 1% penicillin–streptomycin. All cells were cultured at 37 °C in 5% CO_2_.

### RNA isolation and quantitative real-time PCR

Total RNA was extracted using the RNA Purification Kit (EZBioscience, B0004D-100). cDNA was generated with PrimeScript RT Master Mix (Takara, RR036Q). Quantitative real-time polymerase chain reaction (PCR) was performed with Powerup SYBR Green PCR Master Mix (Thermo Fisher Scientific, 1708040) using a QuantStudio 6 Flex Real-Time PCR System (Applied Biosystems, 4484642). All the related real-time PCR primers used in this study are listed in Table S2.

Details are available in Supplementary Materials and Methods.

### Statistical analysis

All statistical analyses were performed using GraphPad Prism 8 software. Data are presented as the means ± SDs. Two-sided Student’s *t* test, unpaired *t* test with Welch’s correction, one-way analysis of variance (ANOVA) with Tukey’s post-test, and 2-way ANOVA with Sidak’s post hoc tests were used for data comparisons, and *P* values less than 0.05 were considered to indicate statistically significant differences.

## Data Availability

All data needed to support the conclusions are presented in the paper or the Supplemental Materials. RNA-seq data have been deposited into the database National Genomics Data Center (https://ngdc.cncb.ac.cn/) (accession number: HRA006815 and CRA015240). The mass spectrometry proteomics data have been deposited to the ProteomeXchange Consortium via the iProX partner repository with the dataset (https://www.iprox.cn/) (accession number: PXD050264). Additional data related to this paper may be requested from the authors.
