# Supplementary material for: LHPP Attenuates Lipid Dysfunction of Uveal Melanoma by Relieving the Histidine Phosphorylation of ACO2
Source: Research (Wash D C). 2025 Sep 22;8:0896. doi: 10.34133/research.0896 (PMC12451111; doi:10.34133/research.0896)
Supplement: Supplementary 1 — Supplementary Materials and Methods Figs. S1 to S6 Tables S1 and S2 [file research.0896.f1.docx]

**Supplementary Materials**

LHPP Attenuates Lipid Dysfunction of Uveal Melanoma by relieving the Histidine Phosphorylation of ACO2

Zhi Yang ^1, 2,*,^ ^†^, Liang Ma ^1, 2,^ ^†^, Yidian Fu ^1, 2,^ ^†^, Xiaoyu He ^1, 2,^ ^†^, Yingxiu Luo ^1, 2,^ ^†^, Shengfang Ge ^1, 2^, Renbing Jia ^1, 2, *^, Jian Huang ^3, *^, Xianqun Fan ^1, 2, *^

^1^ State Key Laboratory of Eye Health, Department of Ophthalmology, Ninth People’s Hospital, Shanghai Jiao Tong University School of Medicine, Shanghai 200025, China

^2^ Shanghai Key Laboratory of Orbital Diseases and Ocular Oncology, Shanghai 200025, China

^3^ Department of Biochemistry and Molecular Cell Biology, Shanghai Key Laboratory of Tumor Microenvironment and Inflammation, Shanghai Jiao Tong University School of Medicine, Shanghai 200025, China

*Address correspondence to: [fanxq@sjtu.edu.cn](mailto:fanxq@sjtu.edu.cn) (X.F.); [jyhuanj@shsmu.edu.cn](mailto:jyhuanj@shsmu.edu.cn) (J.H.); [renbingjia@sjtu.edu.cn](mailto:rengbingjia@sjtu.edu.cn) (R.J.); [yangzhiscience@163.com](mailto:yangzhiscience@163.com) (Z.Y.)

†These authors contributed equally to this work.

**Supplementary Materials**

The supplementary materials are as follows:

**Supplementary Materials and methods**

**Fig. S1-S6**

**Table S1 to S2**

**Supplementary Materials and Methods**

**Subcutaneous and** **intraocular** **xenograft mouse models**

The BALB/c nude mice (male, 6 weeks old) used for the xenograft assay. For the subcutaneous injection model, 1 × 10^6^ melanoma cells from each group were injected subcutaneously (100 μL per mouse) into the left side of the scapular region of BALB/c nude mice (male, 6 weeks old) with a 1 mL sterile syringe (KDL, RWLB). Twenty-eight days after cell injection, the mice were humanely killed for harvesting of tumors. For histological analysis, hematoxylin and eosin (H&E)-stained sections were collected. Tumor volume was calculated with the equation V = ab^2^/2, where a and b are tumor length and width, respectively. Similarly, for the intraocular injection model, BALB/c nude mice were anesthetized, and the sclera was preperforated with a sharp 30-gauge injection needle. A total of 1 × 10^5^ melanoma cells (total volume 5 μL) were intraocularly injected into the choroid of the left eye of BALB/c nude mice (male, 6 weeks old) with a 33-gauge blunt-end sterile microinjection needle (Hamlton,7803-05). Twenty-eight days later, the eyeballs of the mice were collected for H&E and immunostaining analysis.

**Lentivirus packaging for generation of stable cell lines**

To package lentivirus for the generation of stable cell lines, HEK293T cells were seeded in 6-well plates (Corning) containing DMEM supplemented with 10% FBS, with 4x10^5^ cells per well. After 16 hours of incubation and at 70% confluence, the cells were transfected with 0.5 μg of the lentiviral transfer plasmid (LHPP or ACO2), 0.5 μg of pMD2. G (Addgene, 12259), and 1 μg of psPAX2 (Addgene, 12260) in the presence of 6 μL of PolyJet transfection reagent (SignaGen, SL100688) following the manufacturer's protocol. Eight hours after transfection, the medium was replaced with DMEM supplemented with 10% FBS. The viral supernatant was collected after 48 hours, centrifuged at 3000 × g for 15 minutes to remove cellular debris, filtered through a 0.45-mm PVDF filter (Corning), and stored at -80°C for future use. Lentiviral particles were added to 4x10^5^ tumor cells preseeded in 6-well plates (Corning) for transduction in medium containing 10 μg/mL polybrene (Sigma, TR-1003). After 48 hours, the medium was replaced with fresh medium. Cells were selected with 2 μg/mL puromycin (Sangon, E607054) or 10 μg/mL blasticidin (Absin, abs47014830) for 2 weeks. Stable transduction was confirmed by Western blotting.

**Western blotting (WB)**

Cells were harvested at the indicated times and washed three times with PBS. Cell extracts were prepared with RIPA lysis buffer [0.2% SDS (wt/vol), 1% sodium deoxycholate (wt/vol) and 2% NP-40 (vol/vol) in PBS] containing a protease and phosphatase inhibitor cocktail (Invitrogen, 78440). Cell lysates were centrifuged at 12,000 × g for 10 min at 4°C to remove debris. The protein concentration was measured by a BCA protein kit (Beyotime, P0010), and the samples were diluted in SDS‒PAGE sample loading buffer (Beyotime, P0015). Protein samples (~10 μg) were separated by 10% (wt/vol) SDS‒PAGE and transferred to PVDF membranes. After blocking with 5% BSA for 1 hour at room temperature, the membranes were incubated with a primary antibody (2 μg/mL) in antibody diluent buffer (NCM, WB500D) overnight at 4°C. The membranes were then incubated with a secondary antibody conjugated to a fluorescent tag at room temperature for 2 hours. The band signals were visualized and quantified using an Odyssey Infrared Imagining System (LI-COR, Odyssey CLx).

**Immunoprecipitation (IP) of proteins for mass spectrometry**

IP was performed as described previously, with modifications for pHis detection(1)^.^ Stable LHPP-Flag UM cells were lysed in IP buffer [50 mM Tris (pH 8.8), 1 mM EDTA, 100 mM NaCl, 1 mM DTT, 5 mM MgCl_2_, 0.5% NP-40 (vol/vol), and 10% glycerol] containing a protease and phosphatase inhibitor cocktail (Invitrogen, 78440). The samples were centrifuged at 12,000 × g for 10 min at 4°C to remove cell debris. A BCA protein kit (Beyotime, P0010) was then used to measure the protein concentration in each sample. The cell lysates (1 mg) were incubated with anti-Flag magnetic beads (Sigma, M8823) overnight at 4°C. The samples were washed with IP buffer and resuspended in 100 mM triethylamine (TEA; pH 11). The fractions were dried in a SpeedVac and then resuspended in 50 mM Tris-HCl (pH 7.5) for label-free liquid chromatography–tandem mass spectrometry (LC–MS/MS) proteome analysis at the Laboratory of Proteomics, Public Platform for Basic Medicine, Shanghai Jiao Tong University School of Medicine. Additional aliquots of the input and IP eluate supernatants were resuspended in SDS‒PAGE sample loading buffer (Beyotime, P0015) and subjected to WB. LC‒MS/MS data were analyzed and visualized on a bubble diagram with the **SangerBox** tool(2) (<http://www.sangerbox.com/tool>).

**WB for pHis detection**

Because of the instability of pHis under acidic and high-temperature conditions, all steps for pHis signal detection by WB were modified as previously described(3, 4). Unless otherwise stated, all buffers were adjusted to pH 8.8 and precooled at 4°C. The cells were directly lysed in 2× pHis sample buffer (5× pHis sample buffer: 10% SDS, 250 mM Tris-HCl (pH 8.8), 50 mM EDTA, 50% glycerol, 500 mM DTT and 0.02% bromophenol blue). A protease and phosphatase inhibitor cocktail (Invitrogen, 78440) was immediately added to the lysates. The lysates were centrifuged at 12,000 × g for 10 min at 4°C to remove debris. Proteins in unheated clarified lysates were immediately separated at 4°C on a modified SDS‒PAGE gel (stacking gel, pH 8.8; 10% resolving gel, pH 8.8). Lysates heated at 95°C for 10 min to dephosphorylate pHis were used as negative controls for pHis. The bands in the negative heated control lanes represent either nonspecific signals or heat-resistant pHis. The pH 8.8 running and transfer buffers were precooled before use. All steps for electrophoresis (80 V for 30 min and 120 V for 1 hour) and protein transfer (200 mA for 2 hours) were performed at 4°C. The protein-containing PVDF membrane was incubated with blocking buffer (5% BSA, pH 8.8) for 1 hour at 4°C on a shaking platform, and the membrane was then incubated with a primary anti-3-pHis antibody (Creative Diagnostics, CABG-001, rabbit) diluted in antibody diluent buffer (NCM, WB500D) overnight at 4°C. Then, the membrane was washed with 1 × TBST [1 × TBS (pH 8.8) and 0.1% Tween 20] three times (10 min per wash at 4°C) and incubated with a fluorescent tag-conjugated secondary antibody for 2 hours at 4°C.

**IP for pHis detection**

IP was performed as described previously, with modifications for pHis detection [7]. Stable UM cells were lysed in IP buffer [50 mM Tris (pH 8.8), 1 mM EDTA, 100 mM NaCl, 1 mM DTT, 5 mM MgCl_2_, 0.5% NP-40 (vol/vol), and 10% glycerol] supplemented with a protease and phosphatase inhibitor cocktail (Invitrogen, 78440). The samples were centrifuged at 12,000 × g for 10 min at 4°C to remove debris. A BCA protein kit (Beyotime, P0010) was used to measure the protein concentrations in the supernatants. The cell lysates (1 mg) were incubated with anti-HA magnetic beads (Sigma, SAE0197) overnight at 4°C. The samples were washed with IP buffer and resuspended in 2 × pHis sample buffer for WB according to the pHis detection method.

**Cell proliferation assay**

Cell proliferation was evaluated by a CCK-8 assay (MCE, HY-K0301) according to the manufacturer’s instructions. In brief, cells were seeded (5000 cells/100 μL medium) in triplicate in 96-well plates. CCK8 solution was then added to the wells at the indicated time points, and the plates were incubated at 37°C for 2 hours prior to measurement of the absorbance at 450 nm.

**Apoptosis assay**

A FITC-Annexin V Apoptosis Detection Kit I (BD Biosciences, 556547) was used following the manufacturer’s instructions. In brief, a sample containing 5 × 10^5^ cells was centrifuged at 800 × g for 4 min, and the cells were resuspended in 1 mL of PBS, washed twice with cold PBS, stained with FITC-Annexin V and PI on ice for 5 min, and subjected to flow cytometric analysis.

**Cell cycle analysis**

A total of 5 × 10^5^ cells were centrifuged at 800 × g for 4 min, washed twice with PBS, resuspended in 1 mL of PBS and fixed with 75% ethanol overnight at 4°C. The cells were then subjected to FACS at the flow cytometer.

**Colony formation assay**

Equal numbers of UM cells were seeded in a 6-well plate (1000 cells/well in 2 mL of complete medium). The cells were cultured for 3 weeks in DMEM supplemented with 5% FBS. Then, the cells were fixed with formalin and stained with 0.25% crystal violet. The plate was air dried at room temperature, and the colonies were counted.

**Immunohistochemistry (IHC)**

IHC staining of 3-pHis proteins was performed as described previously(5). Here, anti-3-pHis (Creative Diagnostics, CABG-001, rabbit) was used for tissue 3-pHis signal detection. A heated slide was used as the negative control for the 3-pHis signal. After washing with PBS three times, the sections were incubated with secondary antibodies for 2 hours at room temperature and washed three more times with PBS. Nuclei were counterstained with DAPI. Whole-slide images were captured by a panoramic scanner (3DHISTECH, Panoramic MIDI).

**Immunocytochemistry (ICC)**

Approximately 5000 UM cells adhering to a glass slide in a 6-well plate were fixed with 4% formaldehyde for 15 min and then blocked with PBS containing 5% normal goat serum and 0.5% Triton X-100 for 60 min at room temperature. Immunostaining was performed using the appropriate primary antibodies overnight at 4°C. The cellular 3-pHis signal was detected by a purified anti-3-pHis rabbit monoclonal antibody (clone SC 44-8, rabbit, gift from the Tony Hunter laboratory, Salk Institute, USA) generated from SC 44-8 hybridoma cells. A heated slide was treated as the negative control for the 3-pHis signal. The cytoskeleton was counterstained with phalloidin. Nuclei were counterstained with DAPI. Mitochondria were costained with the mitochondrial marker COX-IV. Then, the slides were incubated with the appropriate Alexa Fluor 488- or Alexa Fluor 594-conjugated secondary antibody.

**RNA-seq**

Transcriptome sequencing was conducted by OE Biotech Co., Ltd. (Shanghai, China). Total RNA was isolated from cultured LHPP-overexpressing UM cells or eye tissues of LHPP KO mice (with LHPP WT mice serving as a control) with TRIzol reagent (Invitrogen, 15596026CN). The integrity of the RNA was confirmed with a 2100 Bioanalyzer (Agilent Technologies, USA). The RNA concentration was measured with a Qubit RNA assay kit (Life Technologies, Q32855) and a Qubit 2.0 fluorometer. An Illumina TruSeq RNA Sample Prep Kit (Illumina, RS-122-2001) was used to generate sequencing libraries, which were subsequently sequenced on the Illumina HiSeq 2500 platform. RNA-seq data were analyzed and visualized on a heatmap and a bubble diagram with the SangerBox tool.

**LHPP protein network prediction**

The potential LHPP protein network was predicted by protein–protein interaction network analysis and the functional enrichment analysis database **STRING**(6) (<https://cn.string-db.org/>). Functional enrichment in the LHPP network was further analyzed via a bubble diagram based on the KEGG pathway count and strength and visualized with the SangerBox tool. Subcellular localization analysis of LHPP was performed with the database **COMPARTMENTS(7)** (<https://compartments.jensenlab.org/>), where five-star is the highest confidence and one-star is the lowest.

**Mitochondrial targeting sequence (MTS) prediction and validation**

MTS prediction of human LHPP protein sequences was performed with **iMPL**(8, 9) (<https://csb-imlp.bio.rptu.de/>), which is a tool for predicting additional internal MTS-like signals (iMTS-Ls) in the mature region of proteins that improve the import competence of preproteins and increase the efficiency of their translocation into the mitochondrial matrix. Two putative MTSs of LHPP, one from residues 12 to 56 (MTS1) and one from residues 209 to 235 (MTS2), were identified. Next, MTS1 and MTS2 were fused with GFP individually and cloned and inserted into the Lenti-pCMV-MCS-Flag-Puro vector (Genomeditech, Shanghai). Colocalization of putative MTS-GFP and mitochondria in UM Mum2b cells was visualized with MitoTracker (Invitrogen, M7512). The LHPP mutant lacking the potential MTS (ΔMTS-LHPP) was cloned and inserted into the Lenti-pCMV-MCS-Puro vector (Genomeditech, Shanghai) for subcellular fraction detection.

**Mitochondria isolation**

Mitochondria isolation was performed with a Mitochondria Isolation Kit (Thermo, 89874) according to the manufacturer's instructions. In brief, 2 × 10^6^ UM cells were seeded into 10-cm dishes and cultured in DMEM containing 10% FBS for 48 hours or to 90% confluence. The Mum2b cells were then washed with PBS three times, harvested by trypsin and counted (2 × 10^7^ UM cells was the recommended initial number). Then, 10% of the cells were collected as the “whole cell” fraction, and the remaining 90% of the cells were collected for the next isolation step. The cells were treated with the appropriate lysis reagents and centrifuged at 700 × g for 10 min at 4°C to obtain the pellet, which was considered the nuclear fraction. The remaining supernatant was then centrifuged at 3000 × g for 15 min at 4°C, and the supernatant separated by this step was collected as the cytosolic fraction. The remaining pellet was resuspended and centrifuged at 12,000 × g for 5 min at 4°C, and the resulting pellet was considered the mitochondrial fraction. The protein concentrations in all subcellular fractions were measured with a BCA protein kit. Equal amounts of protein from each fraction were subjected to the next SDS‒PAGE step. The subcellular fractions were subjected to staining with the appropriate internal control antibody: mitochondrial fraction, anti-COX IV (CST, 4850; rabbit); cytosolic fraction, anti-tubulin (CST, 2148; rabbit); and nuclear fraction, anti-histone H3 (CST, 4499; rabbit).

**Analysis of ACO2 PTMs**

PTM sites in the human ACO2 protein (Fig. 6A), including sites of phosphorylation, acetylation and ubiquitylation, were predicted via the mammalian PTM knowledgebase **PhosphoSitePlus(10)** (<https://www.phosphosite.org/>). Classic pHis protein and ACO2 pHis site analyses were performed by **HisPhosSite(11)** (<http://reprod.njmu.edu.cn/hisphossite>). For reanalysis of data for pHis proteins identified by LC‒MS, data for the identified pHis-modified peptides from HeLa cells were downloaded from the latest publication about the histidine phosphoproteome(12). Well-known histidine phosphorylation-related proteins (NME1, NME2, PGAM1, GNB1, and SUCLG1) and the novel identified pHis site and peptide sequences were selected. Specifically, the human ACO2 pHis site (H73) was selectively identified.

**Analysis of protein structure**

The file containing the experimentally determined structure of the human LHPP protein (identifier: 2X4D) was downloaded from the **PDB(13)** (<https://www.rcsb.org/>). The LHPP D17/214 distance and hydrogen bond network were analyzed via **ProteinTools(14)** (<https://proteintools.uni-bayreuth.de/>).

**ATP level measurement**

The ATP levels in UM cell lysates were measured with a CellTiter-Glo ATP assay kit (Promega, G7570) according to the manufacturer’s instructions. In brief, UM cells were lysed in the supplied lysis buffer and centrifuged to remove the insoluble components. The supernatant was then used to isolate mitochondria, and the obtained mitochondrial supernatant was subsequently added to the substrate solution. Luminescence was measured with a luminometer (Promega, GloMax E5311).

**TG level measurement**

Cellular lipid extracts were prepared and dissolved in isopropanol. Cell lysis then was measured by a BCA Protein Quantitation kit (Beyotime, P0010) for normalization. The cellular TG level was measured by commercial Triglyceride Assay kit (Beyotime, S0219S) according to the manufacturer’s instructions.

**Detection of phosphatase activity**

The activity of the phosphatases LHPP and Dead-LHPP was detected by a Phosphatase Assay Kit (Sangon Biotech, C006453) according to the manufacturer’s instructions. Briefly, the concentration of para-nitrophenyl phosphate (pNPP), a chromogenic substrate for most phosphatases, was measured at 405 nm in a 96-well plate.

**Recombinant protein based *in vitro* phosphatase assay**

Briefly, UM Mum2b cells were cultured in FBS-free DMEM in 6-well plates, and 0, 2, 10, or 50 ng/ml recombinant human LHPP protein (Abcam, ab116175) was added for 12 hours. Subsequently, tumor cells were harvested for 3-pHis IF detection or aconitase activity test.

**Transmission electron microscopy**

UM cells were seeded in 6-well plates and fixed with 2.5% isovaleraldehyde overnight at 4°C. After three PBS washes, the samples were incubated with a mixture of 1% osmium tetroxide and 1% potassium ferrocyanide for 2 hours at 4°C. After three additional PBS washes, the samples were dehydrated in a graded ethanol series and subsequently embedded in an epoxy resin. Ultrathin sections (70 nm) were prepared with an ultramicrotome (Leica, EM UC7). Then, the samples were counterstained with uranyl acetate and lead citrate for 10 min each and visualized with a transmission electron microscope (FEI, Tecnai G2 Spirit). Images were acquired with a Veleta digital camera (Olympus Soft Imaging System).

**Histopathology**

After mice were euthanized, mouse tissue samples were obtained and embedded in OCT. The tissues were sliced into sections of an appropriate thickness, and the sections were stained with H&E to evaluate tissue morphology. Cellular lipid droplet accumulation was visualized with an Oil Red O Staining Kit (Servicebio, G1015L) according to the manufacturer's instructions.

**Single-cell transcriptomic analysis**

Patient-derived UM single-cell RNA sequencing analysis was performed as previously described(15). Six UM primary tumor tissue scRNA-seq datasets were acquired from the GEO database (GSE138665). Sample QC were assessed by parameters of nFeature_RNA (100~3000) and percent.mt (no more than 10). Tumor cells were identified using MLANA and MITF. Immune cells were identified by C1QA and CD3D. Endothelial cells were identified by PECAM1 and VWF (16). PRAME was used to label metastatic UM tumor cells. Histidine phosphatase LHPP and kinase NME1 and NME2 were selected for further annotation.

**Citrate concentration measurement**

The cellular citrate concentration was measured with a citrate assay kit (Abcam, ab83396) according to the manufacturer's instructions. In brief, the citrate standard and reaction mixture were prepared in a clear 96-well plate and incubated at room temperature for 30 min in the dark. Finally, the citrate concentration was determined by measuring the optical density (OD) at 570 nm on a colorimetric microplate reader. Similarly, the citrate concentration in mouse eye tissue containing UM tumor cells was measured after tissue homogenization.

**Measurement of ACO2 enzymatic activity**

Mitochondrial aconitase activity was measured with an Aconitase Assay Kit (Abcam, ab83459) following the manufacturer's protocol. In brief, 1 x 10^6^ UM cells were harvested for each assay, washed with cold PBS and resuspended in assay lysis buffer. For mitochondrial aconitase enzymatic activity measurements, cells were permeabilized beforehand with 0.007% digitonin (MedChemExpress, HY-N4000) to remove cytoplasmic proteins as previous reported(17, 18). The cell lysate was centrifuged for 15 minutes at 12,000 × g and 4°C using a microcentrifuge, after which the pellet was collected, resuspended in cold assay buffer, and sonicated for 30 seconds. The protein concentration was measured with a BCA protein kit (Beyotime, P0010). Subsequently, a standard curve and reaction samples were prepared. The reaction mixture was added to a clear 96-well plate and incubated at 25°C for 30 min. Then, development reagent was added, and the plate was incubated at 25°C for 10 min. Finally, the optical density was measured at 450 nm. Damaging effect on activity of aconitase 2 was treated with of exogenously NO induced by 500 μM DETA/NO (MCE, HY-136278). Similarly, the enzymatic activity of ACO2 in mouse eye tissue from xenograft mice was detected after tissue homogenization. UM Mum2b cells were treated by 10 μM recombinant human ACO2 (MedChemExpress, HY-P74432) or ACO2 H73N protein (MedChemExpress, HY- P7S0182) as previous described(19).

**KEGG metabolic pathway analysis**

The *Homo sapiens* (human) metabolic pathway information was obtained from **KEGG** (<https://www.kegg.jp/kegg-bin/show_pathway?hsa01100>). LHPP controls three-way crosstalk among metabolic pathways: lipid metabolism, histidine metabolism and the TCA cycle.

**References**

1. Hindupur SK*, et al.* (2018) The protein histidine phosphatase LHPP is a tumour suppressor. *Nature* 555(7698):678-682.

2. Shen W*, et al.* (2022) Sangerbox: A comprehensive, interaction-friendly clinical bioinformatics analysis platform. *iMeta* 1(3):e36.

3. Fuhs SR*, et al.* (2015) Monoclonal 1- and 3-Phosphohistidine Antibodies: New Tools to Study Histidine Phosphorylation. *Cell* 162(1):198-210.

4. Kalagiri R, Adam K, & Hunter T (2020) Empirical Evidence of Cellular Histidine Phosphorylation by Immunoblotting Using pHis mAbs. *Methods Mol Biol* 2077:181-191.

5. Luhtala N & Hunter T (2020) Immunohistochemistry (IHC): Chromogenic Detection of 3-Phosphohistidine Proteins in Formaldehyde-Fixed, Frozen Mouse Liver Tissue Sections. *Methods Mol Biol* 2077:193-208.

6. Szklarczyk D*, et al.* (2023) The STRING database in 2023: protein-protein association networks and functional enrichment analyses for any sequenced genome of interest. *Nucleic Acids Res* 51(D1):D638-D646.

7. Binder JX*, et al.* (2014) COMPARTMENTS: unification and visualization of protein subcellular localization evidence. *Database (Oxford)* 2014:bau012.

8. Backes S*, et al.* (2018) Tom70 enhances mitochondrial preprotein import efficiency by binding to internal targeting sequences. *J Cell Biol* 217(4):1369-1382.

9. Boos F, Muhlhaus T, & Herrmann JM (2018) Detection of Internal Matrix Targeting Signal-like Sequences (iMTS-Ls) in Mitochondrial Precursor Proteins Using the TargetP Prediction Tool. *Bio Protoc* 8(17):e2474.

10. Hornbeck PV*, et al.* (2015) PhosphoSitePlus, 2014: mutations, PTMs and recalibrations. *Nucleic Acids Res* 43(Database issue):D512-520.

11. Zhao J*, et al.* (2021) HisPhosSite: A comprehensive database of histidine phosphorylated proteins and sites. *J Proteomics* 243:104262.

12. Cui F, Qian X, & Ying W (2021) Integrated Strategy for Unbiased Profiling of the Histidine Phosphoproteome. *Anal Chem* 93(47):15584-15589.

13. Burley SK*, et al.* (2023) RCSB Protein Data Bank (RCSB.org): delivery of experimentally-determined PDB structures alongside one million computed structure models of proteins from artificial intelligence/machine learning. *Nucleic Acids Res* 51(D1):D488-D508.

14. Ferruz N, Schmidt S, & Hocker B (2021) ProteinTools: a toolkit to analyze protein structures. *Nucleic Acids Res* 49(W1):W559-W566.

15. Pandiani C*, et al.* (2021) Single-cell RNA sequencing reveals intratumoral heterogeneity in primary uveal melanomas and identifies HES6 as a driver of the metastatic disease. *Cell Death Differ* 28(6):1990-2000.

16. Durante MA*, et al.* (2020) Single-cell analysis reveals new evolutionary complexity in uveal melanoma. *Nat Commun* 11(1):496.

17. Drapier JC & Hibbs JB, Jr. (1986) Murine cytotoxic activated macrophages inhibit aconitase in tumor cells. Inhibition involves the iron-sulfur prosthetic group and is reversible. *J Clin Invest* 78(3):790-797.

18. Palmieri EM*, et al.* (2020) Nitric oxide orchestrates metabolic rewiring in M1 macrophages by targeting aconitase 2 and pyruvate dehydrogenase. *Nat Commun* 11(1):698.

19. Mansilla S*, et al.* (2023) Redox sensitive human mitochondrial aconitase and its interaction with frataxin: In vitro and in silico studies confirm that it takes two to tango. *Free Radic Biol Med* 197:71-84.


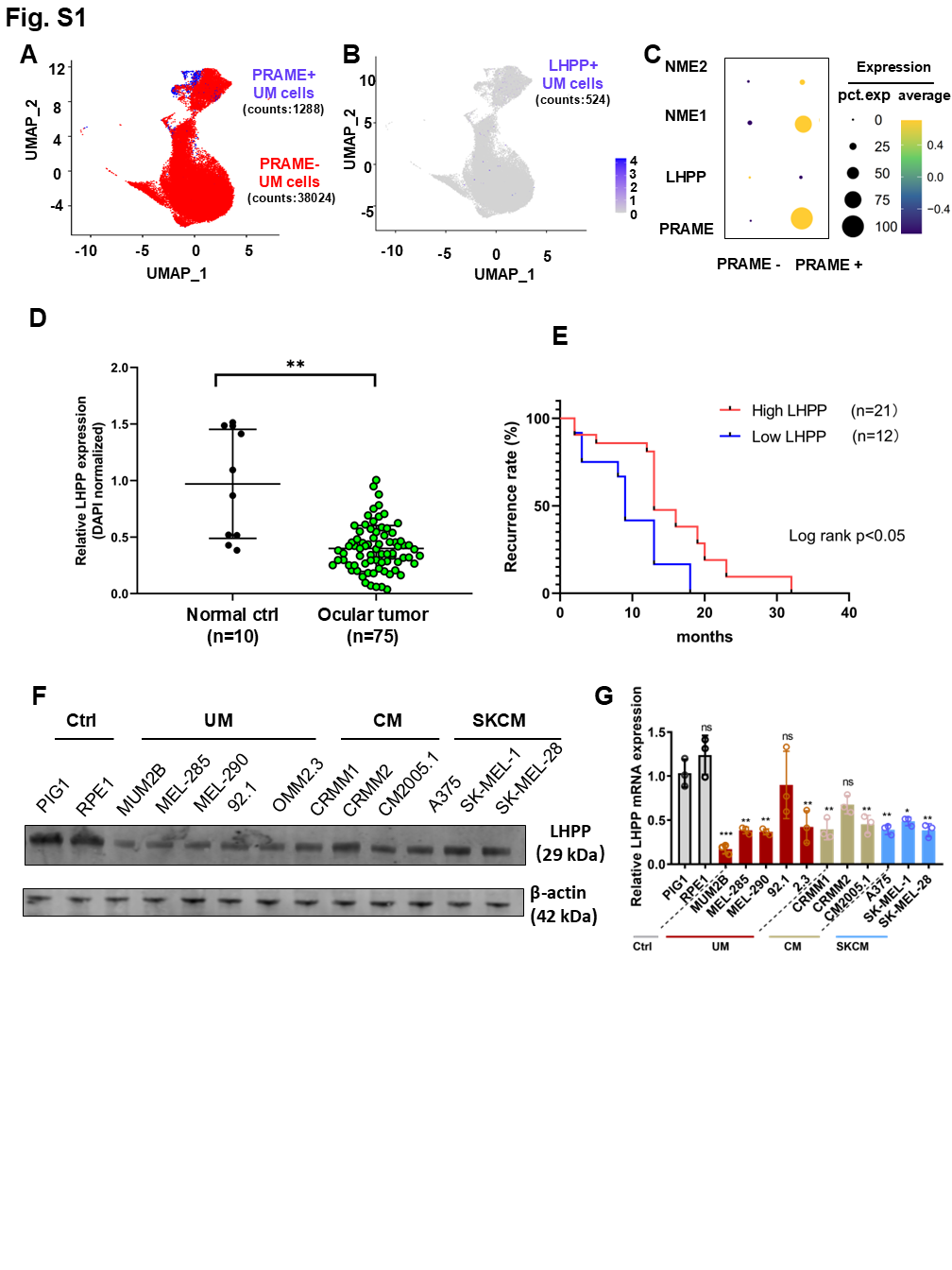
 **Fig. S1, related to Fig. 1.** LHPP is a downregulated marker in UM.

**(A)** Single-cell RNA-seq U-map plot of 39312 identified UM tumor cells including 1288 PRAME-positive (blue) and 38024 PRAME-negative (red) UM tumor cells, and **(B)** 524 LHPP-positive (purple) UM tumor cells. **(C)** Bubble map showing the expression of PRAME, LHPP, NME1 and NME2 in the PRAME-positive or negative UM tumor cell cluster. **(D)** Quantification of LHPP in human normal ctrl tissues (n=10) and human ocular melanoma tissues (n=75) based on IF analysis. Unpaired t test with Welch's correction. **p < 0.01. **(E)** Kaplan‒Meier curves of tumor recurrence showing the difference between ocular melanoma patients with low (n=12) and high (n=21) LHPP expression levels. Log-rank test, p < 0.05. **(F)** LHPP protein level and **(G)** LHPP mRNA level in normal control cells, UM cells, CM cells and SKCM cells. One-way ANOVA with Tukey’s post hoc test. **p* <0.05, ** *p* <0.01, *** *p* <0.001.


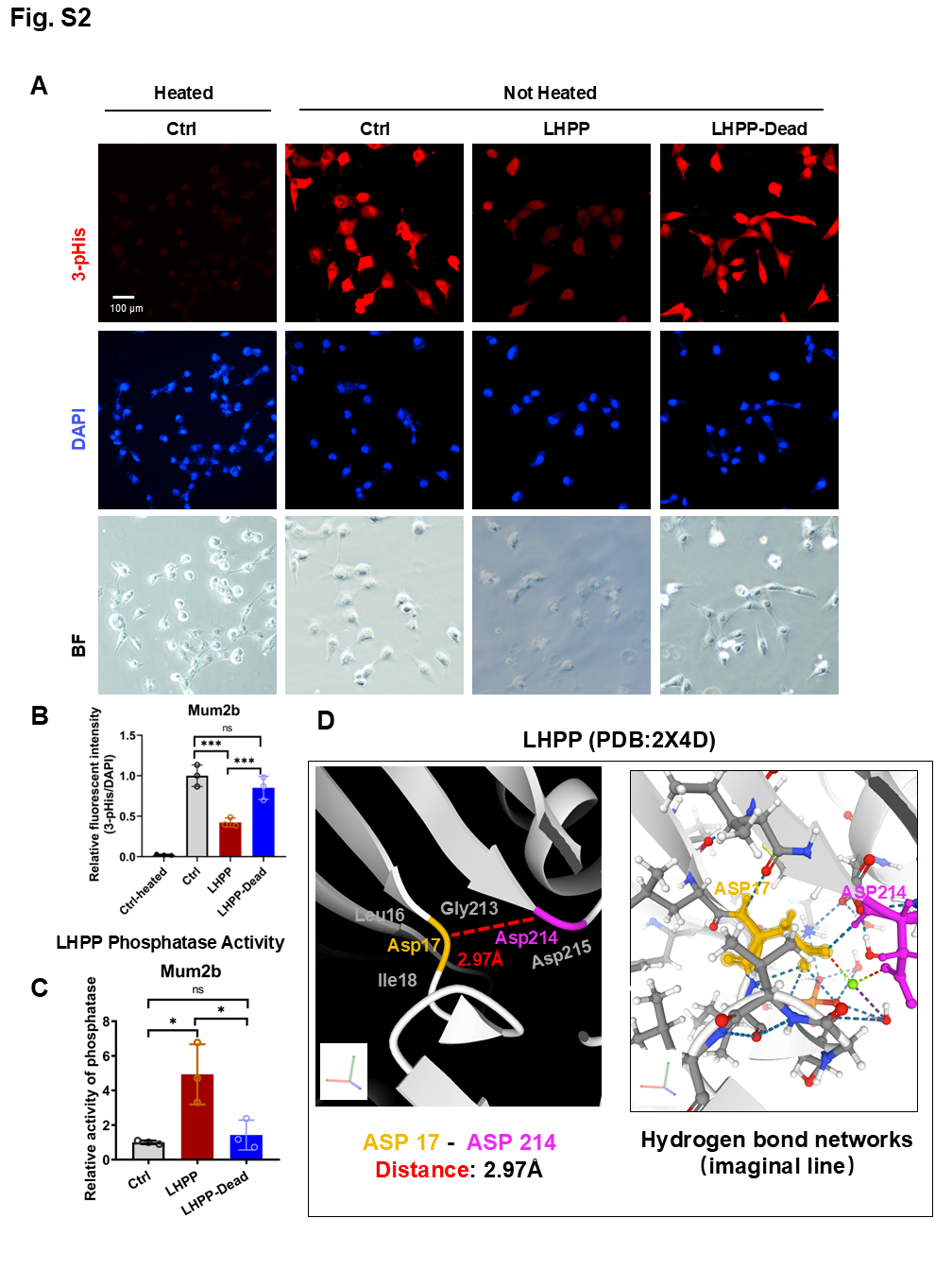
**Fig. S2, related to Fig. 2.** LHPP decreases global 3-pHis level in UM Mum2b cells.

**(A)** Representative image of IF staining of 3-pHis in tumors in UM Mum2b cells expressing LHPP or LHPP-Dead. Scale bar: 100 μm. **(B)** Quantification of the relative level of 3-pHis (normalized to DAPI). n = 3. Data are presented as the means ± SDs. Two-tailed unpaired Student’s t test. ***p < 0.001. **(C)** Phosphatase activity comparison between LHPP and LHPP-Dead. n = 3. Data are presented as the means ± SDs. Two-tailed unpaired Student’s t test. *p < 0.05. **(D)** Protein structure of human LHPP (PDB ID: 2X4D) and potential hydrogen bond network between LHPP ASP17 and LHPP ASP214.


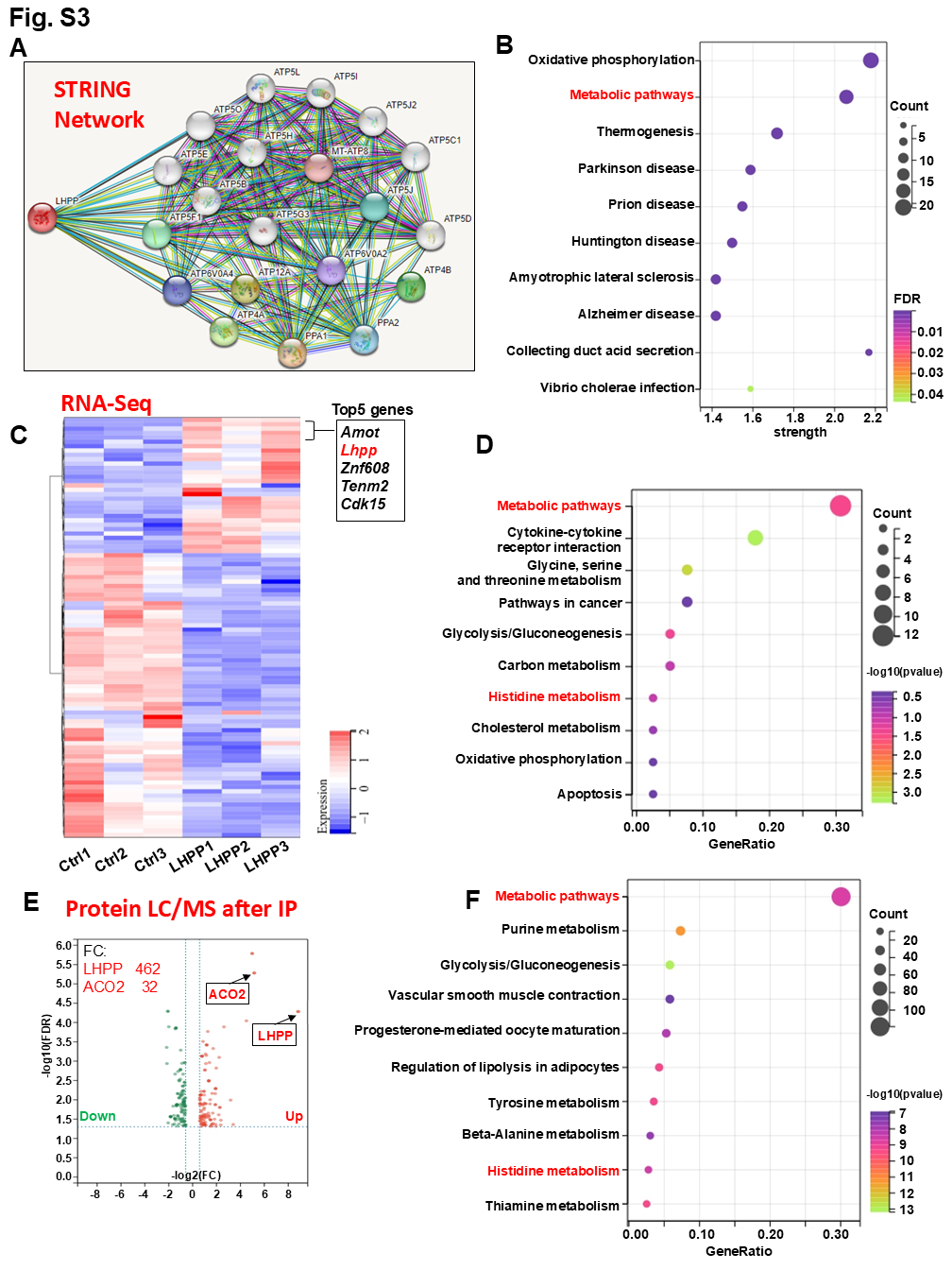


**Fig. S3.** **related to Fig. 3.** Multi-omics reveal LHPP as a metabolism-related protein

**(A)** Potential LHPP-interacting proteins were predicted via the **STRING** database. **(B)** Functional enrichment of the potential LHPP interaction network based on the KEGG pathway count and strength. **(C)** Heatmap of genes differentially expressed between LHPP-overexpressing and control Mum2b cells identified by RNA-seq. The top 5 upregulated genes are highlighted, and LHPP (ranked 2^nd^) is highlighted in red. **(D)** KEGG analysis of genes differentially expressed by RNA-seq. **(E)** Volcano plot: altered proteins identified by mass spectrometry after IP of LHPP-Flag in stable UM Mum2b cells. **(F)** KEGG analysis of proteins differentially expressed between LHPP-Flag-overexpressing and control Mum2b cells identified by LC‒MS/MS after IP with an anti-Flag antibody.


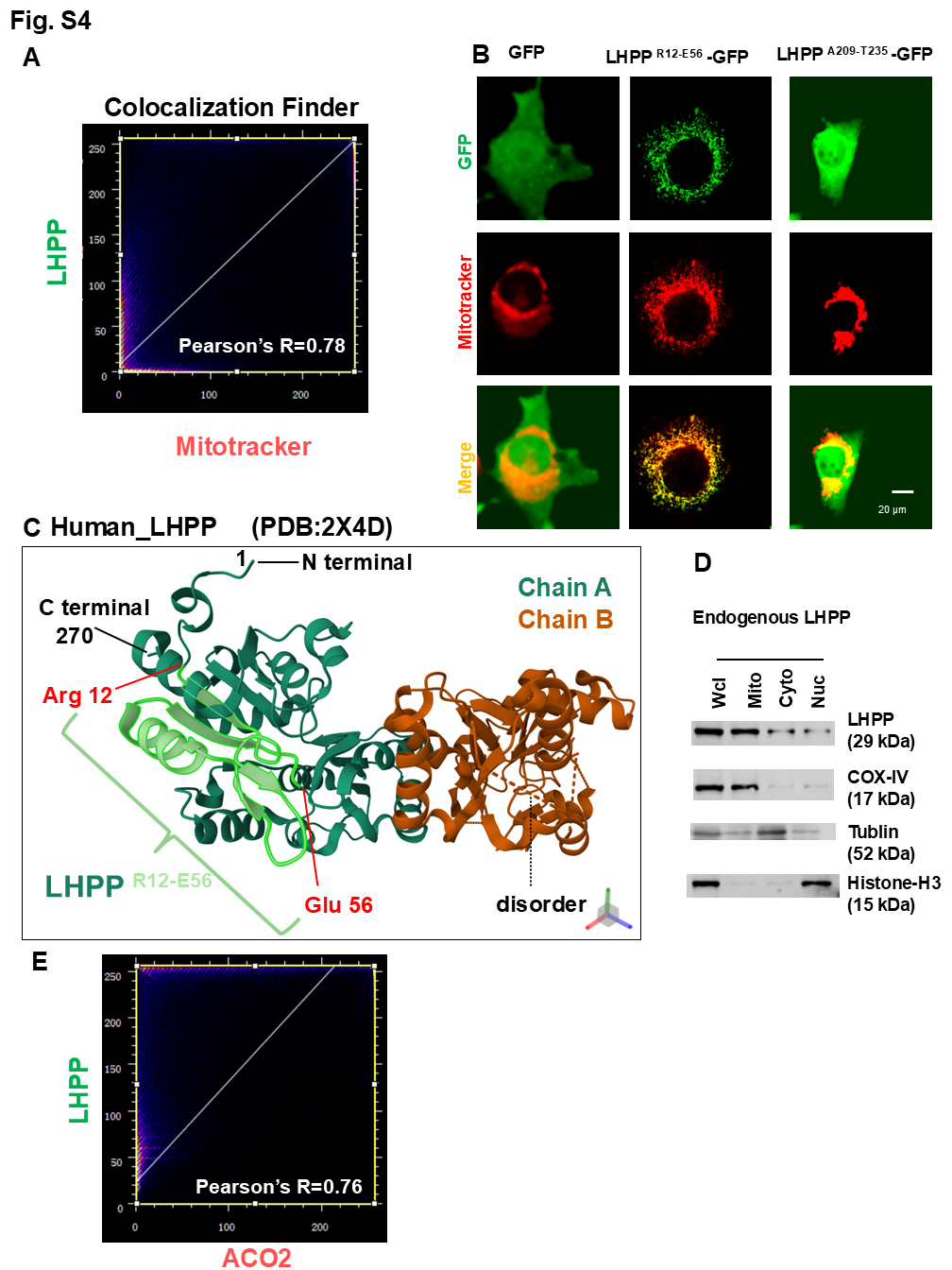


**Fig. S4, related to Fig. 3**. Identification of potential LHPP MTS.

**(A)** Scatter Plot: Colocalization analysis of LHPP and MitoTracker by Image J plugin Colocalization Finder. Pearson’s R=0.78. **(B)** Co-localization of GFP and mitochondrial marker MitoTracker fluorescence signal in Mum2b cells expressing wildtype GFP, LHPP 12-56 -GFP and LHPP 209-235 -GFP. **(C)** Protein structure analysis showing LHPP R12-E56 sequences locating in the surface of LHPP protein. **(D)** Subcellular location of endogenous LHPP protein in UM Mum2b cell line**. (E)** Colocalization analysis of LHPP and ACO2. Pearson’s R=0.76.


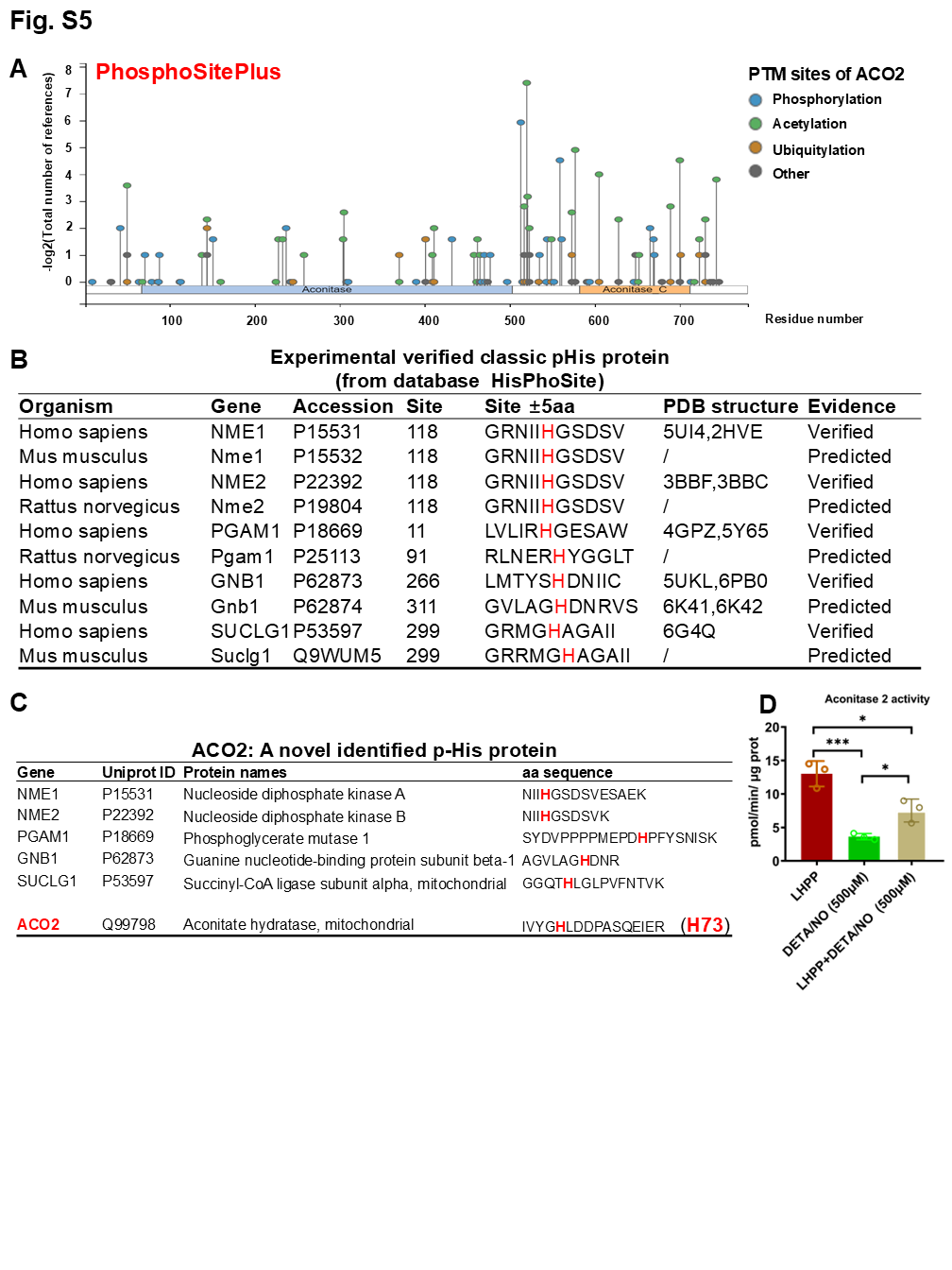


**Fig. S5, related to Fig. 4.** Validation of the pHIs site of human ACO2 protein.

**(A)** **PhosphoSitePlus**: Analysis of ACO2 PTM sites, including phosphorylation, acetylation and ubiquitylation sites. **(B)** Experimentally verified classical pHis-related proteins and the predicted pHis sites. Data were retrieved from **HisPhosSite**. **(C)** pHis proteins identified by LC‒MS/MS, including classical histidine phosphorylation proteins and ACO2. **(D)** Activity of aconitase in UM Mum2b cells stably overexpressing LHPP, treated with DETA/NO (500 μM), LHPP combined with DETA/NO. n=3. One-way ANOVA with Tukey’s post hoc test. *p < 0.05, ***p<0.001.


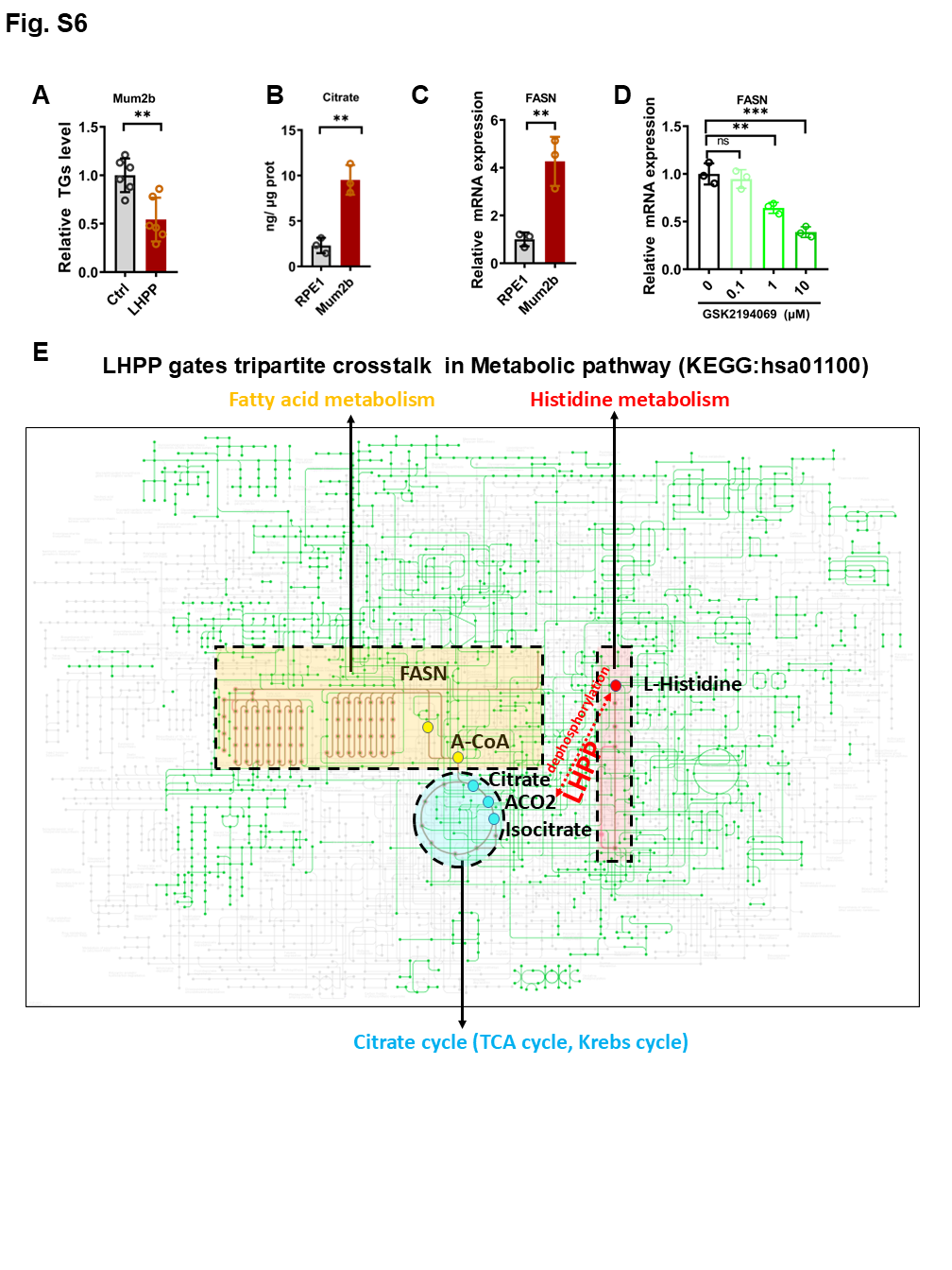


**Fig. S6, related to Fig. 6.** Potential role of LHPP in lipid metabolism.

**(A)** Relative TGs level in Mum2b Ctrl and Mum2b cells with stable LHPP overexpression. Two-tailed unpaired Student’s t test. **p < 0.01. n=6. **(B)** Relative FASN mRNA expression in UM Mum2b cells and **(C)** normal human retinal pigment epithelium RPE1 cells. Two-tailed unpaired Student’s t test. **p < 0.01. n=3. (**D**) Relative human FASN mRNA expression after treated with 0, 0.1, 1, 10 μM fatty acid synthase inhibitor GSK2194069 in UM Mum2b cells. Data were analyzed by one-way ANOVA with Tukey’s post hoc test. **p<0.01, ***p<0.001. n=3. **(E)** LHPP controls three-way crosstalk among metabolic pathways: fatty acid metabolism, histidine metabolism and the TCA cycle.

| **Table.S1.** Clinical characteristics of involved UM tissues | | | | |  |
| --- | --- | --- | --- | --- | --- |
| **Array ID** | **Sex (Female=0, Male=1)** | **Age (Year)** | **T Stage** | **Recurrence  (Month)** |  |
| A1 | 1 | 61 | 3 | 3 |  |
| A2 | 1 | 61 | 3 | 3 |  |
| A3 | 0 | 63 |  |  |  |
| A4 | 0 | 58 | 2 |  |  |
| A5 | 0 | 58 | 2 |  |  |
| A6 | 0 | 58 | 2 |  |  |
| A7 | 1 | 31 | 3 | 13 |  |
| A8 | 1 | 31 | 3 | 13 |  |
| A9 | 1 | 32 | 2 |  |  |
| A10 | 1 | 32 | 2 |  |  |
| A11 | 1 | 32 | 2 |  |  |
| A12 | 1 | 21 | 2 |  |  |
| B1 | 1 | 21 | 2 |  |  |
| B2 | 1 | 21 | 2 |  |  |
| B3 | 0 | 66 | 2 |  |  |
| B4 | 0 | 66 | 2 |  |  |
| B5 | 0 | 66 | 2 |  |  |
| B6 | 0 | 69 | 2 |  |  |
| B7 | 0 | 31 | 2 | 3 |  |
| B8 | 0 | 31 | 2 | 3 |  |
| B9 | 1 | 65 | 4 |  |  |
| B10 | 1 | 65 | 4 |  |  |
| B11 | 0 | 67 | 2 | 0 |  |
| B12 | 0 | 67 | 2 | 0 |  |
| C1 | 1 | 23 | 2 |  |  |
| C2 | 1 | 23 | 2 |  |  |
| C3 | 1 | 23 | 2 |  |  |
| C4 | 1 | 52 | 2 | 0 |  |
| C5 | 0 | 1 |  |  |  |
| C6 | 0 | 1 |  |  |  |
| C7 | 0 | 1 |  |  |  |
| C8 | 1 | 12 | 2 |  |  |
| C9 | 1 | 12 | 2 |  |  |
| C10 | 1 | 73 | 2 | 13 |  |
| C11 | 1 | 73 | 2 | 13 |  |
| C12 | 1 | 73 | 2 | 13 |  |
| D1 | 1 | 75 | 2 | 0 |  |
| D2 | 1 | 75 | 2 | 0 |  |
| D3 | 0 | 52 | 3 |  |  |
| (Continued on next page) | | | | | |
|  | | | | |  |
| Continued |  |  |  |  |  |
| **Array ID** | **Sex (Female=0, Male=1)** | **Age (Year)** | **T Stage** | **Recurrence  (Month)** |  |
| D4 | 0 | 52 | 3 |  |  |
| D5 | 1 | 31 | 3 | 13 |  |
| D6 | 1 | 78 | 2 | 0 |  |
| D7 | 1 | 78 | 2 | 0 |  |
| D8 | 0 | 8 |  |  |  |
| D9 | 0 | 8 |  |  |  |
| D10 | 0 | 29 |  |  |  |
| D11 | 0 | 42 | 3 | 18 |  |
| D12 | 0 | 42 | 3 | 18 |  |
| E1 | 1 | 66 | 3 | 20 |  |
| E2 | 1 | 66 | 3 | 20 |  |
| E3 | 0 | 29 |  |  |  |
| E4 | 1 | 58 | 2 | 0 |  |
| E5 | 1 | 58 | 2 | 0 |  |
| E6 | 0 | 82 | 3 |  |  |
| E7 | 0 | 82 | 3 |  |  |
| E8 | 0 | 82 | 3 |  |  |
| E9 | 0 | 53 | 2 | 5 |  |
| E10 | 1 | 80 | 2 |  |  |
| E11 | 1 | 73 | 2 |  |  |
| E12 | 1 | 60 | 3 | 9 |  |
| F1 | 1 | 60 | 3 | 9 |  |
| F2 | 1 | 60 | 3 | 9 |  |
| F3 | 1 | 3 |  |  |  |
| F4 | 0 | 51 | 3 | 12 |  |
| F5 | 0 | 58 | 2 |  |  |
| F6 | 0 | 58 | 2 |  |  |
| F7 | 1 | 52 | 2 | 2 |  |
| F8 | 1 | 52 | 2 | 2 |  |
| F9 | 1 | 73 | 2 | 13 |  |
| F10 | 1 | 73 | 2 | 13 |  |
| F11 | 1 | 73 | 2 | 13 |  |
| F12 | 1 | 81 | 2 | 0 |  |
| G1 | 1 | 31 | 3 | 13 |  |
| G2 | 0 | 26 |  |  |  |
| G3 | 1 | 20 | 3 |  |  |
| G4 | 1 | 20 | 3 |  |  |
| G5 | 1 | 20 | 3 |  |  |
| G6 | 1 | 14 |  |  |  |
| G7 | 1 | 74 | 3 | 0 |  |
| G8 | 1 | 74 | 3 | 0 |  |
| G9 | 1 | 74 | 3 | 0 |  |
| G10 | 1 | 47 | 3 | 16 |  |
| G11 | 1 | 47 | 3 | 16 |  |
| (Continued on next page) | | | | | |
| Continued | | | | |  |
| **Array ID** | **Sex (Female=0, Male=1)** | **Age (Year)** | **T Stage** | **Recurrence  (Month)** |  |
| G12 | 0 | 77 | 3 | 19 |  |
| H1 | 0 | 77 | 3 | 19 |  |
| H2 | 1 | 49 | 2 | 0 |  |
| H3 | 1 | 54 | 2 | 2 |  |
| H4 | 1 | 67 | 3 | 0 |  |
| H5 | 1 | 63 | 3 | 32 |  |
| H6 | 1 | 63 | 3 | 32 |  |
| H7 | 1 | 38 | 3 | 8 |  |
| H8 | 1 | 70 | 3 | 23 |  |
| H9 | 1 | 70 | 3 | 23 |  |
| H10 | 1 | 70 | 3 | 23 |  |

| **Table.S2.** Primers used for qPCR in this study | |
| --- | --- |
| **Primer name** | **Sequences** |
| **m-*LHPP*-F-qPCR** | GACATCTCCGGGGTGCTATG |
| **m-*LHPP*-R-qPCR** | CTTTCAGCGGGGACTGTTTCA |
| **m-*ACO2*-F-qPCR** | ATCGAGCGGGGAAAGACATAC |
| **m-*ACO2*-R-qPCR** | TGATGGTACAGCCACCTTAGG |
| **m-*FASN*-F-qPCR** | GGAGGTGGTGATAGCCGGTAT |
| **m-*FASN*-R-qPCR** | TGGGTAATCCATAGAGCCCAG |
| **m-*ACTB*-F-qPCR** | GTGACGTTGACATCCGTAAAGA |
| **m-*ACTB*-R-qPCR** | GCCGGACTCATCGTACTCC |
|  |  |
| **h-*LHPP*-F-qPCR** | GAGGCTGGGATTTGACATCTC |
| **h-*LHPP*-R-qPCR** | GAGCAGGTATGGTCGCAGG |
| **h-*ACO2*-F-qPCR** | CCCTACAGCCTACTGGTGACT |
| **h-*ACO2*-R-qPCR** | TGTACTCGTTGGGCTCAAAGT |
| **h-*FASN*-F-qPCR** | TTCTACGGCTCCACGCTCTTCC |
| **h-*FASN*-R-qPCR** | [GAAGAGTCTTCGTCAGCCAGGA](https://mail.163.com/js6/read/readhtml.jsp?mid=26:1tbiGggVTlaEEPxTgQAAsl&userType=ud&font=15&color=3370FF) |
| **h-*ACTB*-F-qPCR** | CACCATTGGCAATGAGCGGTTC |
| **h-*ACTB*-R-qPCR** | AGGTCTTTGCGGATGTCCACGT |
